# A dual organelle-targeting mechanosensitive probe

**DOI:** 10.1126/sciadv.abn5390

**Published:** 2023-01-11

**Authors:** Po-Yu Ho, Tsu Yu Chou, Chuen Kam, Wenbin Huang, Zikai He, Alfonso H. W. Ngan, Sijie Chen

**Affiliations:** ^1^ Ming Wai Lau Centre for Reparative Medicine, Karolinska Institutet, Hong Kong, P. R. China.; ^2^ School of Science, Harbin Institute of Technology, Shenzhen, HIT Campus of University Town, Shenzhen 518055, P. R. China.; ^3^ Department of Mechanical Engineering, The University of Hong Kong, Pokfulam Road, Hong Kong, P. R. China.

## Abstract

Cells are responsive to the mechanical environment, but the methods to detect simultaneously how different organelles react in mechanobiological processes remain largely unexplored. We herein report a dual organelle-targeting fluorescent probe, (*E*)-1-[3-(diethoxyphosphoryl)propyl]-4-[4-(diethylamino)styryl]pyridin-1-ium bromide (ASP-PE), for mechanical mapping in live cells. ASP-PE is aggregation-induced emission active and is sensitive to the local mechanical environment. It targets the plasma membrane (PM) and intracellular mitochondria in cells by its phosphonate moiety and pyridinium. In this work, through ASP-PE staining, changes of membrane tension in the PM and mitochondria in response to varied osmotic pressure and substrate stiffness are visualized using fluorescence lifetime imaging microscopy. The mechanobiological importance of actin filaments and microtubules in the PM and mitochondria is also investigated using this probe. Computational simulations are applied to study the sensing mechanism of the probe. This study introduces a unique tool for mapping the membrane tension in the PM and mitochondria together, providing us great opportunities to study organelle’s interactions in mechanobiology.

## Teaser

Advanced fluorescent AIE probe enables mapping of the mechanical dynamics of cell membrane and mitochondria.

## INTRODUCTION

Mechanical forces exerted by the extracellular environment are counterbalanced by intracellular pressure and play important roles in cellular physiology and homeostasis. The impacts of these biophysical stimuli are evident in dictating cell fate and behaviors, and mechanical cues in the cellular microenvironment have been found important in many biological processes such as cancer progression (*1*). For example, mechanical fluctuations in the cellular microenvironment, including tensile stress (*2*), hydrostatic pressure (*3*), shear stress (*4*), interfacial geometry (*5*), and matrix stiffness (*6*), are shown influencing and governing carcinogenesis (*1*, *7*). Understanding the relationships between mechanobiology and cancers can thus lead to the development of new diagnostic methods and therapeutical interventions. Micropipette aspiration and atomic force microscope cantilevers (or optical/magnetic tweezers) are the most commonly used techniques for evaluating cell mechanical properties or imaging mechanobiological events (*8*). These methods can provide quantitative measurements. However, they require experienced operators. Moreover, only the mechanical property of a single target can be assessed in each single measurement, and the intracellular compartments are not directly accessible for these contact-based methods. Therefore, convenient methods rendering spatialtemporal information of mechanical properties in multiple organelles are highly desirable in the field of mechanobiology to complement the current tools and to provide information that is inaccessible using the current methods.

The plasma membrane (PM) is the foremost and pivotal interface that mediates cell mechanobiological responses to external mechanical stimuli (*9*). For example, an environment of high stiffness induces the metastatic phenotype mechanotransduced by integrin (*6*, *10*). To better understand the mechanobiology in live cells, several fluorescence imaging techniques focusing on the PM have been exploited, such as fluorescence recovery after photobleaching, fluorescence resonance energy transfer, and Laurdan imaging (*11*–*13*). In addition to the PM, other intracellular organelles, especially mitochondria, are also important targets in mechanobiological studies. In particular, how mitochondrial dynamics and biogenesis are modulated in response to mechanical stimuli during epithelial-mesenchymal transition and metastasis have been intensively studied (*4*, *14*–*17*).

Recently, Matile *et al.* (*18*–*21*) have reported a series of dithienothiophene-based fluorescent membrane tension (σ) probes that locally sense one designated organelle membrane each time in live cells. Although mechanical stress can be transmitted from the PM to intracellular organelles, tools to unveil simultaneously how the PM and intracellular organelles respond to mechanical stress are highly desirable but not yet available. Along with the development of conventional bioprobes, fluorescent probes that exhibit aggregation-induced emission (AIE) have emerged rapidly in the field of bioimaging. AIE-active fluorophores emit weakly or are even nonemissive in molecularly dissolved state with active intramolecular motions, but they emit intensively when the intramolecular motion is restricted (*22*–*24*). This mechanism, named restriction of intramolecular motion (RIM), has been used to develop various fluorescent probes (*25*).

Here, we designed and synthesized a mechanosensitive dual PM-mitochondria–targeting AIE-active fluorescent probe named ASP-PE. Unlike the existing tools that track only the PM but no other intracellular compartments simultaneously, ASP-PE is a bioprobe that specifically targets both the PM and mitochondria at the same time. We demonstrated that ASP-PE could detect single-cell mechanotype from its fluorescence lifetime (τ_f_) in the PM and mitochondria under external mechanical stimuli including tension, compression, and changes in substrate stiffness. Distinct mechanotype changes in the PM and mitochondria under different mechanical disturbances were revealed by ASP-PE. The role of cytoskeletons in cellular mechanobiology was also demonstrated using ASP-PE. Molecular dynamics (MD) investigation coupled to a stimulated phospholipid bilayer structure was used to investigate the relationship between membrane tension and fluorescence lifetime of the fluorescent probes in silico. Structural comparisons among ASP-PE and two styryl dyes suggested that the phosphonate moiety of ASP-PE was critical for its PM targetability and high mechanosensitivity in the target membrane.

## RESULTS

### Molecular design and photophysical properties of ASP-PE

**Fig. 1.**
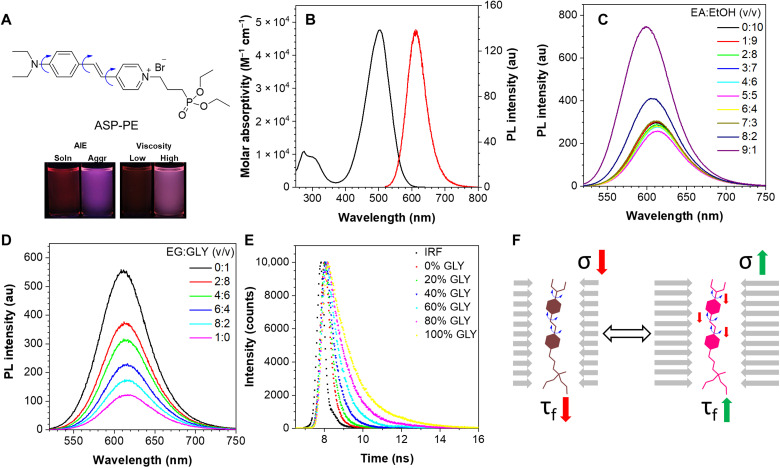
Chemical structure and photophysical properties of ASP-PE. (**A**) Chemical structure of ASP-PE. The blue arrows indicate the key rotatable bonds. Insets: Fluorescence images of different binary solutions in the presence of ASP-PE (10 μM). Left: EA:ethanol (EtOH) [0:1 versus 9:1 (v/v)]. Right: ethylene glycol (EG) versus GLY. AIE, aggregation-induced emission. (**B**) UV-Vis absorption (black line) and PL (red line) spectra of ASP-PE (10 μM) in EtOH. au, arbitrary units. (**C**) PL spectra of ASP-PE (10 μM) in the solution mixtures of EA and EtOH (in different volume ratios). (**D**) PL spectra of ASP-PE (10 μM) in the solution mixtures of EG and GLY (in different volume ratios). All PL spectra were measured at a 515-nm photoexcitation. (**E**) TCSPC spectra of ASP-PE (10 μM) in the mixture of EG and GLY with different volume ratios at 293 K. The fitting algorithm analysis calculates the fluorescence lifetime(s) of 0, 20, 40, 60, 80, and 100% GLY solution mixtures, and they are reported to be 0.35, 0.42, 0.59, 0.84, 1.12, and 1.44 ns, respectively. IRF, instrumental response function. (**F**) Schematic illustration of the mechanosensitive indicator in the PM and other membranous organelles based on the fluorescence lifetime imaging microscopy (FLIM) signal.

The new mechanosensitive probe ASP-PE (molecular structure shown in Fig. 1A) was synthesized from commercially available chemical reagents through a straightforward two-step synthetic route as shown in fig. S1 (see the synthetic details in the Supplementary Materials). ASP-PE is constituted of an electron push-pull π-conjugation skeleton. It is soluble in common organic solvents such as dichloromethane, methanol, ethanol, and dimethyl sulfoxide (DMSO) but insoluble in most hydrophobic solvents, such as hexane, diethyl ether, and ethyl acetate (EA). The intermediate (*E*)-*N*,*N*-diethyl-4-(2-[pyridin-4-yl)vinyl]aniline and ASP-PE were structurally characterized by ^1^H- and ^13^C-nuclear magnetic resonance (NMR) spectroscopy as well as high-resolution mass spectrometry (HRMS) (figs. S2 to S4). The photophysical properties of ASP-PE were studied and illustrated in Fig. 1. ASP-PE exhibited an absorption peak at the wavelength (λ_abs_) of 500 nm with a molar absorptivity (ε) of ca. 5 × 10^4^ M^−1^ cm^−1^ and an emission peak (λ_em_) at ca. 610 nm in ethanol solution (10 μM; photoexcitation at 515 nm) (Fig. 1B). Its absorption and emission spectra in different single solvents are given in fig. S5. When the EA fraction of the solution(s) increased beyond 0.7, ASP-PE tended to reach the aggregated state due to the lower solvation power of solvent mixtures. An enhanced photoluminescence (PL) signal with a minor and hypsochromic shift (ca. 10 nm) could be found in the solutions with aggregates [i.e., EA:EtOH = 8:2 or 9:1 (v/v)], demonstrating an AIE characteristic (Fig. 1C). In a viscous environment with a high glycerol (GLY) fraction, the probe exhibited stronger fluorescence and longer fluorescence lifetime under confinement (Fig. 1, D and E).

### Biocompatibility and dualorganelle targetability of ASP-PE in live cells

**Fig. 2.**
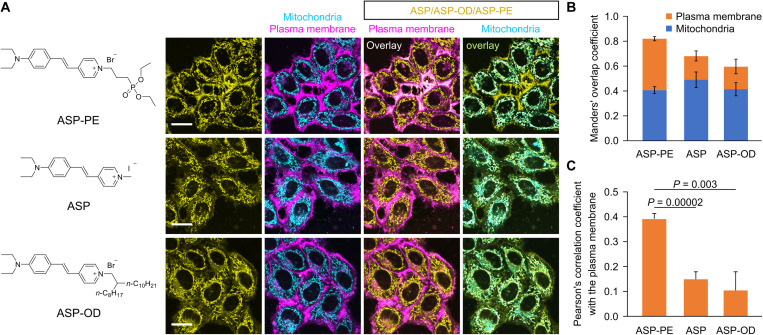
The dual localization of ASP-PE in the PM and mitochondria. (**A**) Confocal images of the costaining of ASP-PE (10 μM), ASP (10 μM), or ASP-OD (10 μM) with the PM probe (CellMask Deep Red Plasma Membrane Stain) and the mitochondria probe (BioTracker 405 Blue Mitochondria Dye) in HeLa cells. Scale bars, 20 μm. (**B**) Overall colocalization with the PM and mitochondria was analyzed by the Manders’ overlap coefficient. Data represent means ± SD. *n* = 4 independent experiments. (**C**) Colocalization of ASP-PE, ASP, or ASP-OD with the PM was analyzed by the Pearson’s correlation coefficient. Data represent means ± SD. *n* = 4 independent experiments. Exact *P* values are shown. Statistical differences were determined by a one-way ANOVA with Tukey’s post hoc test.

The cytotoxicity of ASP-PE was evaluated using the Cell Counting Kit-8 (CCK-8) assay before bioapplication. As illustrated in fig. S6, the cell viability was more than 70% at a concentration below 15 μM, demonstrating a good biocompatibility of ASP-PE in vitro. We then tested its performance in live-cell imaging by incubating HeLa cells with 10 μM ASP-PE for 15 min. ASP-PE specifically labeled the cell surface and intracellular compartments. Costaining experiments showed that these signals were colocalized with the commercial PM and the mitochondria probes (CellMask Deep Red Plasma Membrane Stain and BioTracker 405 Blue Mitochondria Dye, respectively) (Fig. 2A). Overall, ~80% of the ASP-PE signal overlaid with the PM and mitochondria (Fig. 2B), revealing that the dual targetability of ASP-PE to the PM and mitochondria was highly selective.

To understand the chemical structure of ASP-PE that attributes to this dual targeting specificity, we experimentally compared ASP-PE with two structurally similar styryl dyes (structures shown in Fig. 2A). The first one is (*E*)-4-[4-(diethylamino)styryl]-1-methylpyridin-1-ium iodide (ASP) with one methyl group locking to the pyridinium ring, which is reported as a mitochondrial probe for live-cell imaging (*26*). The second one is (*E*)-4-[4-(diethylamino)styryl]-1-(2-octyldodecyl)pyridin-1-ium bromide (ASP-OD) with a long and branched alkyl chain on the pyridinium, which was structurally characterized by ^1^H- and ^13^C-NMR spectroscopy as well as HRMS (figs. S7 to S9). The photophysical properties of ASP and ASP-OD were depicted in the ultraviolet-visible (UV-Vis) absorption spectra (fig. S10), PL spectra (figs. S10 and S11), and time-correlated single-photon counting (TCSPC) spectra (fig. S12). These data showed that all three molecules have similar photophysical properties because of the same π-conjugation moiety. In the fluorescence imaging, ASP preferentially labeled the mitochondria in HeLa cells (Fig. 2A). With the long and branched side chain, ASP-OD targeted to the mitochondria and weakly outlined the cell boundary (Fig. 2A). By comparing the subcellular distribution, ASP-PE exhibited the best PM and mitochondria localization among the above three molecules (Fig. 2, B and C). We inititally postulated that the electronegative diethyl phosphonate moiety in ASP-PE mimics the negatively charged phospholipid head, which provides better PM retention than the hydrophobic interactions between the branched alkyl chain in ASP-OD and the fatty acid tails of phospholipids. Meanwhile, the pyridinium in ASP-PE, as in ASP and ASP-OD, allows its mitochondrial localization. These, therefore, confer ASP-PE with dual targetability to the PM and mitochondria.

### Distinct τ_f_ of ASP-PE in PM and mitochondria and its sensitivity to osmotic pressure

**Fig. 3.**
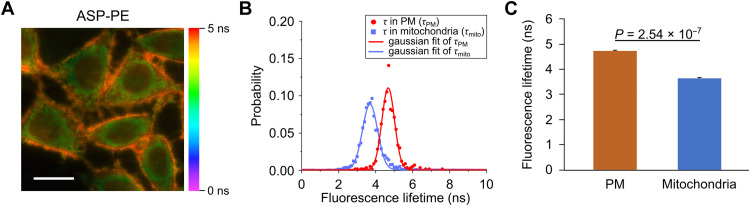
The distinct fluorescence lifetimes of ASP-PE in the PM and mitochondria. (**A**) Fluorescence lifetime image of ASP-PE in the PM and mitochondria of HeLa cells. Scale bar, 20 μm. (**B**) Fluorescence lifetime distribution of ASP-PE in the PM and mitochondria with Gaussian fitting. (**C**) Quantification of ASP-PE lifetime in the PM and mitochondria. Data represent means ± SEM. *n* = 4 independent experiments. Statistical differences were determined by two-tailed Student’s *t-test*. Exact *P* values are shown.

The average ASP-PE lifetime in the PM and mitochondria were distinguishable under fluorescence lifetime imaging microscopy (FLIM) at 4.73 and 3.64 ns, respectively (Fig. 3). In comparison, the mean τ_f_ of ASP and ASP-OD, which did not have the (diethoxyphosphoryl)propyl moiety, was measured in cells (figs. S13 and S14). The mean τ_f_ of ASP in the PM and mitochondria was 4.19 and 2.65 ns, respectively, whereas the mean τ_f_ of ASP-OD in the PM and mitochondria was 1.55 and 1.28 ns, respectively. Their τ_f_ ranges or mean τ_f_ differences in the PM and mitochondria were not substantial when comparing to ASP-PE. 

**Fig. 4.**
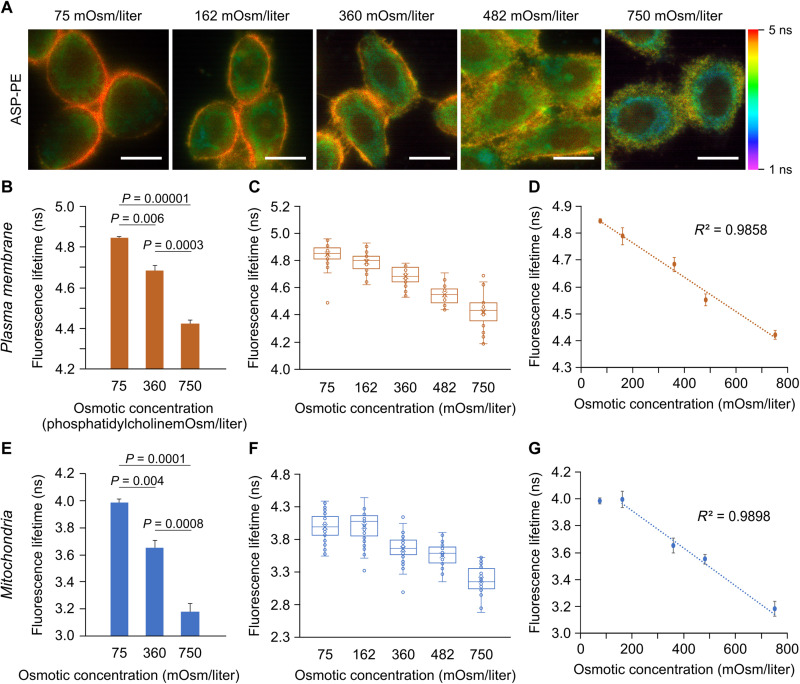
ASP-PE senses the changes of mechanical properties in the PM and mitochondria caused by transient osmotic shocks. (**A**) Fluorescence lifetime of ASP-PE in HeLa cells treated with culture medium with different osmotic concentrations. Scale bars, 20 μm. (**B** to **G**) Quantification of ASP-PE fluorescence lifetime in hypotonic medium (75 mOsm/liter), isotonic medium (360 mOsm/liter), or hypertonic medium (750 mOsm/liter) in the PM (B to D) and mitochondria (E to G). Data represent means ± SEM. *n* = 4 independent experiments. Exact *P* values are shown. Statistical differences were determined by a one-way ANOVA with Tukey’s post hoc test (B and E). Box and whisker plot display fluorescence lifetime of ASP-PE in individual cells (C and F). ASP-PE fluorescence lifetime showed two linear relationships with osmotic concentration in the PM from 75 to 750 mOsm/liter and in mitochondria from 162 to 750 mOsm/liter (D and G).

It is reported that osmotic challenge induced by the hypotonic condition, in which the osmolarity was lower than iso-osmolarity (360 mOsm/liter), causes tensile stress, whereas the hypertonic condition causes compressive pressure (*27*). To determine whether ASP-PE lifetime is sensitive to changes in mechanical properties, osmotic shock was applied to ASP-PE–stained cells. By using FLIM, ASP-PE showed opposite fluorescence lifetime changes in HeLa cells challenged with hypotonic (75 mOsm/liter) or hypertonic (750 mOsm/liter) solutions when comparing with the isotonic control (Fig. 4A). The mean τ_f_ of ASP-PE in the PM increased significantly under hypotonic shock, but it decreased upon hypertonic treatment (Fig. 4, B and C). The mean τ_f_ of ASP-PE in the PM was inversely proportional to the osmolarity of the incubation buffer with the *R*^2^ (coefficient of determination) of 0.9858 (Fig. 4D). The linear correlation of the τ_f_ of ASP-PE in the PM under varied osmotic stress indicates a primary mechanobiological change in the PM, which is possibly caused by altered membrane metrics such as membrane tension and lipid phase separation (*18*, *28*, *29*). Moreover, we also observed a similar phenomenon for the mean τ_f_ of ASP-PE in mitochondria under both hypoosmotic and hyperosmotic conditions as compared with the isotonic condition (Fig. 4, A, E, and F). A linear correlation of the mean τ_f_ of ASP-PE as a function of osmolarity was found with the *R*^2^ of 0.9898 from 162 to 750 mOsm/liter (Fig. 4G). Regarding to ASP (fig. S13) and ASP-OD (fig. S14), their fluorescence lifetimes were less sensitive to osmotic shock. So, the floppy moiety of diethyl phosphonate in ASP-PE acts like a “mechanosensitivity enhancer” toward the membranous organelles without alternating the π-conjugation skeleton of the fluorescent unit. As a result, ASP-PE can be used to sense changes of mechanical properties in the PM and mitochondria.

### Varied substrate stiffness alters PM tension

The cellular microenvironment in vivo is complex and dynamic with stiffness heterogeneity that induces cellular adaptation (*1*, *6*). For example, the metastatic behavior of mammary cancer cells can be influenced by matrix stiffness (*30*). The importance of substrate-dependent mechanobiological response prompts us to further examine whether ASP-PE can detect these influences with varied substrate stiffness.

**Fig. 5.**
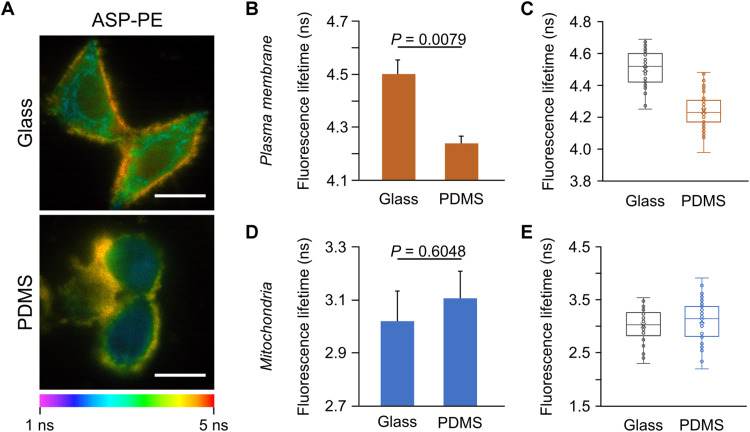
The effects of different substrate stiffness on the membrane tension of HeLa cells. (**A**) Representative FLIM images of ASP-PE–stained HeLa cells seeded on the glass or PDMS surface. Both substrates were precoated with collagen I. Scale bars, 20 μm. (**B** to **E**) Quantification of ASP-PE fluorescence lifetime in the PM (B and C) and mitochondria (D and E). Softer PDMS surface resulted in a shorter τ_f_ of ASP-PE in the PM but not in the mitochondria. Data represent means ± SEM. *n* = 4 independent experiments. Statistical differences were determined by two-tailed Student’s *t-test*. Exact *P* values are shown (B and D). Box and whisker plots display fluorescence lifetime of ASP-PE in individual cells (C and E).

To fabricate substrates with softer stiffnesses compared to the glass, commercially available polydimethylsiloxane (PDMS; SYLGARD 184) was used. Its mechanical property was revealed by its stress-strain curve where Young’s moduli was 855 kPa for the PDMS. In addition, the thickness of these PDMS thin films was controlled to be around 90 μm. It was thicker than the critical value of 38 μm which is the marginal thickness for cells responding to substrate stiffness (*31*). Cells were seeded on the PDMS surface and a glass-bottom culture dish, respectively, for comparison. Under FLIM, HeLa cells seeded on PDMS substrates showed a rounder morphology, and their lifetime maps of ASP-PE were different to those seeded on the glass surface with a Young’s modulus in gigapascals (Fig. 5A). For cells grown on the PDMS substrate, the mean τ_f_ of ASP-PE in the PM decreased significantly compared with cells grown on the glass surface (Fig. 5, B and C). On the other hand, the mean τ_f_ of ASP-PE in mitochondria showed no significant difference among them (Fig. 5, D and E). Provided that adherent cells can sense and respond to substrate stiffness so that stiffer substrates result in more rigid cells with higher tension overall (*32*), our data are consistent with that and demonstrate that ASP-PE lifetime can sense changes in PM tension upon varied substrate stiffness from kilopascals to gigapascals.

### The relation of the cytoskeleton and membrane tension is revealed by ASP-PE

The cytoskeleton is a highly dynamic protein network that provides cells with structural support, a framework for organelle movement, and a system for generation of mechanical forces. The fence and picket model proposes that actin filaments extensively tether to the PM that confines the lateral movement of molecules in the membrane *(**9*, *33**)*. This lateral organization structurally strengthens the PM against external forces. On the other hand, microtubules are involved in maintenance of cell shape, cell adhesion, as well as organelle positioning and trafficking.

**Fig. 6.**
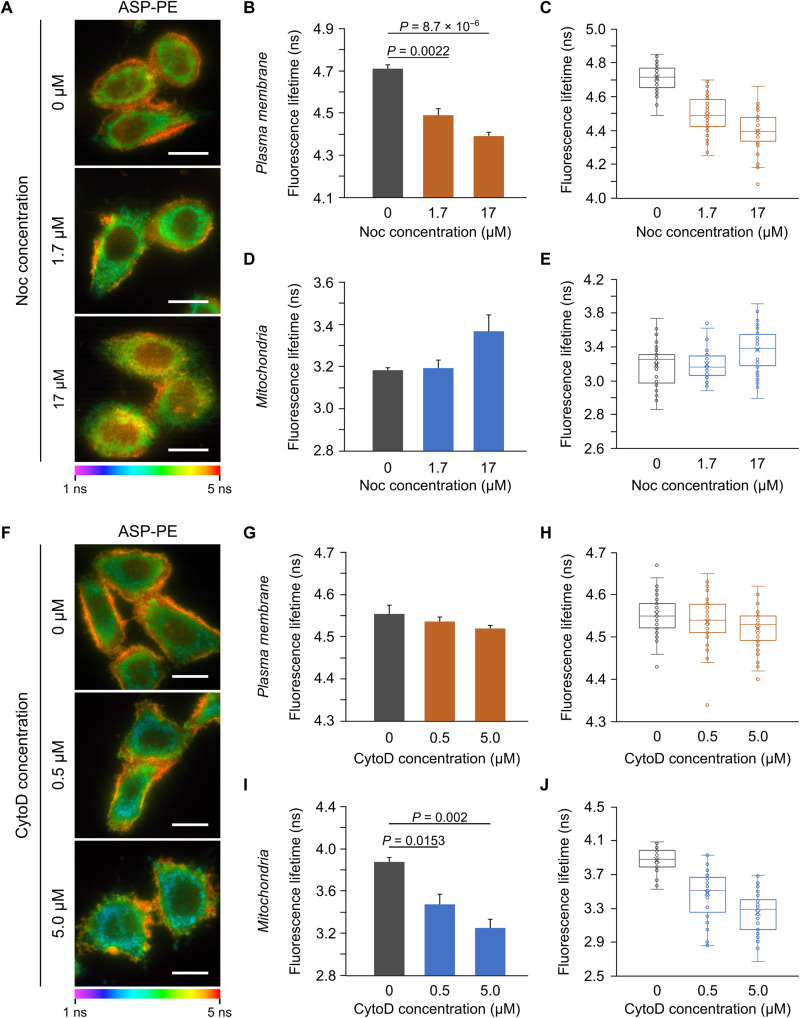
The importance of cytoskeleton on the mechanobiology of the PM and mitochondria. (**A**) Fluorescence lifetime of ASP-PE in the PM and mitochondria of HeLa cells treated with Noc. Scale bar, 20 μm. (**B** to **E**) Quantification of ASP-PE fluorescence lifetime in the PM (B and C) and mitochondria (D and E) of untreat and Noc-treated cells. Data represent means ± SEM. *n* = 4 independent experiments. Exact *P* values are shown. Statistical differences were determined by a one-way ANOVA with Tukey’s post hoc test (B and D). Box and whisker plots display fluorescence lifetime of ASP-PE in individual cells (C and E). (**F**) Fluorescence lifetime of ASP-PE in the PM and mitochondria of HeLa cells treated with CytoD. Scale bars, 20 μm. (**G** to **J**) Quantification of ASP-PE fluorescence lifetime in the PM (G and H) and mitochondria (I and J) of untreat and CytoD-treated cells. Data represent means ± SEM. *n* = 4 independent experiments. Exact *P* values are shown. Statistical differences were determined by a one-way ANOVA with Tukey’s post hoc test (G and I). Box and whisker plots display fluorescence lifetime of ASP-PE in individual cells (H and J).

To study the importance of the cytoskeleton in mechanobiology, we destabilized microtubules and actin filaments by nocodazole (Noc) and cytochalasin D (CytoD), respectively (*34*, *35*). Fluorescence imaging showed that microtubules were fragmented by Noc (fig. S15A), while networks of actin filaments were disassembled by CytoD (fig. S15B). We then investigated the τ_f_ difference of ASP-PE in the PM and mitochondria under the pharmacological treatments. Under FLIM, disorganization of microtubules decreased the mean τ_f_ of ASP-PE in the PM of Noc-treated cells (Fig. 6, A to C) but not significantly changed ASP-PE lifetime in mitochondria (Fig. 6, D and E). This implies that cell contraction due to microtubule disruption decreases the PM tension. Conversely, disassembly of actin filaments had no significant effect on the mean τ_f_ of ASP-PE in the PM of CytoD-treated cells (Fig. 6, F to H) but decreased ASP-PE lifetime in mitochondria (Fig. 6, I and J). This suggests that cell shrinkage following actin depolymerization exerted increased intracellular stress on mitochondrial membrane, leading to a decreased membrane tension in the mitochondrial membrane.

### MD simulation investigation

#### MD simulations of probe-membrane interactions

**Fig. 7.**
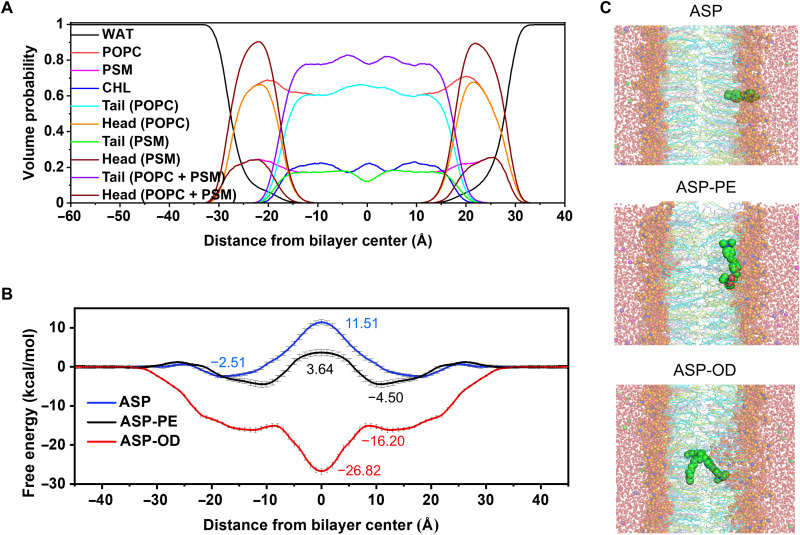
Structure of the simulated phospholipid bilayer and MD of embedded staining styryl dyes. (**A**) Volume probability profiles of the equilibrated phospholipid bilayer consisted of POPC, CHOL, and PSM phospholipids in water (WAT). (**B**) Free-energy profiles of translocation of the ASP, ASP-PE, and ASP-OD molecules from the center of the phospholipid membrane to the water phase. In addition, the free energy was set to zero in the water phase. The SDs were also shown. (**C**) Global snapshots of ASP, ASP-PE, and ASP-OD in the phospholipid bilayer [ASP (top) in the position 18 Å from the center of the phospholipid bilayer, ASP-PE (middle) in the position 11 Å from the center of the phospholipid bilayer, and ASP-OD (bottom) in the position 0 Å from the center of the phospholipid bilayer]. The phosphatidylcholine head groups of POPC phospholipids and the sphingomyelin head groups of PSM phospholipids were shown as orange spheres. The palmitoyl acyl chains and oleoyl acyl chains of POPC phospholipids were shown as cyan sticks and limon sticks, respectively. The palmitoyl acyl chains and sphingosine acyl chains of PSM phospholipids were shown as cyan sticks and pink sticks, respectively. The CHOL phospholipids were shown as light blue sticks. The ASP, ASP-PE, and ASP-OD molecules were shown as green spheres. Meanwhile, the neutralizing K^+^ ions and Cl^−^ ions were shown as purple spheres and magenta spheres, respectively. The water molecules were shown as red spheres.

To gain further insight into the probing mechanism, MD simulation was used to unravel the relationship between the structures of staining agents and their mechanical sensitivities toward the environment of phospholipid bilayers. Before determining the free-energy profiles of translocation of the ASP, ASP-PE, and ASP-OD molecules from the center of the phospholipid bilayer to the water phase, equalized phospholipid membranes consisted of 1-palmitoyl-2-oleoyl-*sn*-glycero-3-phosphocholine (POPC) phospholipids, cholesterol (CHOL), and *N*-palmitoyl-*d*-*erythro*-sphingosylphosphorylcholine (PSM) phospholipids were first obtained by the MD simulations (Fig. 7A). On the basis of the equalized phospholipid membrane, three phospholipid membrane models with their phospholipid membrane centers occupied by the ASP, ASP-PE, and ASP-OD molecules, respectively, were built (fig. S16). Last, the pulling MD simulations were used to pull the three molecules from the center of the phospholipid membranes to the water phase (fig. S17), and the resultant simulation windows along the pulling paths were further used to perform the bias MD simulation with umbrella sampling to sampling. The overlap degree between the histogram distributions of any two adjacent windows was used to evaluate the adequacy of sampling (fig. S18).

On the basis of the sampled data from all simulation windows and the weighted histogram analysis method techniques (*36*), the relative free-energy profiles of translocation of ASP, ASP-PE, and ASP-OD molecules from the center of the phospholipid membrane to the water phase were presented in Fig. 7B. The phospholipid membrane center and the water phase served as the reference of *Z* position as 0 Å and free-energy shifted to 0 kcal/mol, respectively. For the entry of ASP and ASP-PE molecules to the phospholipid membrane, their free-energy profiles dropped to −2.51 and −4.50 kcal/mol, respectively, after an almost negligible energy barrier and reached to the global minimum state. However, comparing to the *Z* position of ASP molecule (*Z* = 18 Å) at its global minimum, the *Z* position of ASP-PE molecule (*Z* = 11 Å) was closer to the phospholipid membrane center. From the global minimum to the global maximum in the phospholipid membrane center (*Z* = 0 Å), both the free-energy profiles of ASP and ASP-PE molecule needed to undergo a continuous endothermic process to overcome the energy barrier. For the entry of ASP-OD molecule to the phospholipid membrane, its free-energy profile first dropped to −16.20 kcal/mol and reached a minimum at *Z* = 12 Å. After overcoming an almost negligible energy barrier, its free-energy profile further dropped sharply until it reached the global minimum state in the phospholipid membrane center (*Z* = 0 Å) whose free-energy was −26.82 kcal/mol. From the simulation, it is shown that although these three molecules have the same π-conjugation moiety, they present different stable positions (i.e., global minimum) and translocation abilities (i.e., the energy barrier that had to overcome from the water phase to the phospholipid membrane center) in the phospholipid membrane. The calculated effective resistance coefficients and effective permeability coefficients are shown in table S1.

To unravel the intrinsic mechanism for the differences of the most stable positions and translocation abilities of ASP, ASP-PE, and ASP-OD molecules, interactions between these three molecules and the phospholipid membrane in their most stable positions were analyzed. As shown in Fig. 7C, when the ASP molecule was in the most stable position (*Z* = 18 Å), it inserted vertically into the phospholipid membrane with its positively charged pyridine ring surrounded by the negatively charged phosphatidylcholine/sphingomyelin head groups. This insertion mode could enhance the electrostatic interaction between these two groups. However, when the ASP-PE molecule was in the most stable position (*Z* = 11 Å), it laid parallel to the phospholipid membrane with its positively charged pyridine ring was in proximity to but not surrounded by the negatively charged phosphatidylcholine/sphingomyelin head groups (shown in Fig. 7C). This parallel orientation could be attributed to the diethyl phosphate group next to the pyridine ring, which, to a certain extent, played a role in electrostatic repulsion between the ASP-PE molecule and phosphatidylcholine/sphingomyelin head groups.

The phospholipid membrane center was the most stable position (*Z* = 0 Å) of the ASP-OD molecule (shown in Fig. 7C). This arrangement may be related to the presence of hydrophobic *n*-octyl and *n*-decyl chains next to the pyridine ring. It enhanced the hydrophobic interaction between the ASP-OD molecule and the alkyl chains in the phospholipid membrane, which allows complete anchoring of the ASP-OD molecule. This postulation is consistent with the negatively charged phosphatidylcholine/sphingomyelin head groups that are in proximity to the positively charged pyridine ring of the ASP-OD molecule. From the above analysis, we concluded that it was the structural differences of the photo-inactive moiety among ASP, ASP-PE, and ASP-OD molecules that determined their most stable positions and postures in the phospholipid membrane.

#### Fluorescence properties of ASP, ASP-PE, and ASP-OD

Besides the most stable positions and translocation abilities, fluorescence properties of ASP, ASP-PE, and ASP-OD molecules were also simulated and calculated. On the basis of the conformation of ASP, ASP-PE, and ASP-OD molecules in the pure ethanol solvent and the phospholipid membrane randomly extracted from umbrella sampling structures at the window with the largest partition (i.e., the most stable position; fig. S19), their structural changes, electronic excitation properties, and reorganization energies were used to determine the fluorescence properties. Notably, different membrane tensions (+50, 0, and −200 dyne/cm) were considered for each probe conformation in the phospholipid membrane during the quantitative calculation of fluorescence properties.

First, the structural changes between the ground state (S_0_ state) and the first excited singlet state (S_1_ state) were considered. As shown in fig. S20, the root mean square deviations (RMSD; an indicating value showing the degree of similarity or superimposition of conformations) between S_0_ state and S_1_ state for the ASP, ASP-PE, and ASP-OD molecules in the pure ethanol solvent were 0.067, 0.111, and 0.138 Å, respectively. When these molecules were embedded in the phospholipid membrane with a surface tension of +50 dyne/cm, the RMSDs for the ASP, ASP-PE, and ASP-OD molecules were 0.049, 0.088, and 0.087 Å, respectively. When the surface tension of phospholipid membrane was changed to 0 dyne/cm, the corresponding RMSD values decreased to 0.037, 0.067, and 0.065 Å, respectively. When the surface tension of phospholipid membrane was −200 dyne/cm, they further decreased to 0.030, 0.024, and 0.037 Å, respectively. Obviously, the phospholipid membrane could reduce the conformational fluctuations of ASP, ASP-PE, and ASP-OD molecules and increase their rigidity. The more order the phospholipid membrane is, the smaller fluctuations and more rigid of the embedded dyes will be (fig. S21).

Second, the reorganization energies (RE) of S_0_ state and S_1_ state were further considered. As shown in fig. S20, when the ASP, ASP-PE, and ASP-OD molecules translocate from the pure ethanol solvent to phospholipid membranes, all their RE decreased from 21 to 24 kcal/mol to 4 to 6 kcal/mol. Moreover, the more ordered phospholipid membrane resulted in the lower RE of the embedded ASP, ASP-PE, and ASP-OD molecules. Combining the results of structural changes and reorganization energies, it was shown that the ASP, ASP-PE, and ASP-OD molecules in the pure ethanol solvent underwent relatively large structural changes upon photoexcitation because of the high freedom of intramolecular motion. This leads to high RE to recover the structural changes caused by the molecular motion. However, when the dye molecules were embedded in the phospholipid membrane, their freedom of molecular motion naturally decreases due to the densely packing nature of the phospholipid membrane. Therefore, during the electron excitation from S_0_ to S_1_ state, the ASP, ASP-PE, and ASP-OD molecule only underwent relatively small structural motions and with lower RE to recover the conformations of the ground state.

Considering that the recovery of structural rotations primarily goes through the nonradiative transition, the nonradiative decay rate constants of membrane-embedded ASP, ASP-PE, and ASP-OD molecules will decrease due to the limitation of motion. This was confirmed by the calculated nonradiative decay rate constant for ASP-PE molecule as shown in Table 1 and table S2. Meanwhile, the decrease of nonradiative decay rate constant upon the increased membrane tension causes the higher quantum yield and longer fluorescence lifetime.

**Table 1.** Calculated photophysical properties of ASP-PE. Calculated absorption data, emission data, transition orbital assignments of the optimized S_1_ states, radiative rate constant, nonradiative rate constant, and fluorescence lifetime for ASP-PE. HOMO, highest occupied molecular orbital; LUMO, lowest unoccupied molecular orbital.

Absorption (nm)	*f*_abs_	Emission (nm)	*f*_em_	Assignment of S_1_	Radiative rate constant (s^−1^)	Nonradiative rate constant (s^−1^)	Fluorescence lifetime (ns)
In ethanol solution							
369.50	1.5486	606.26	1.8657	HOMO-1 → LUMO (2.50%)	3.40 × 10^8^	1.22 × 10^11^	0.01
				HOMO→LUMO (93.07%)			
In phospholipid bilayer membrane (surface tension = +50 dyne/cm)							
406.06	1.4187	500.15	1.6707	HOMO-1 → LUMO (5.09%)	4.44 × 10^8^	5.63 × 10^9^	0.16
				HOMO→LUMO (88.91%)			
				HOMO→LUMO+2 (2.53%)			
In phospholipid bilayer membrane (surface tension = 0 dyne/cm)							
410.86	1.5151	482.30	1.5679	HOMO-1→LUMO (6.20%)	4.50 × 10^8^	2.56 × 10^9^	0.33
				HOMO→LUMO (87.16%)			
				HOMO→LUMO+2 (3.08%)			
In phospholipid bilayer membrane (surface tension = −200 dyne/cm)							
413.68	1.5122	449.55	1.4913	HOMO-1→LUMO (6.02%)	4.93 × 10^8^	1.07 × 10^9^	0.64
				HOMO→LUMO (88.18%)			
				HOMO→LUMO+2 (2.39%)			

For ASP-PE molecule, when the surface tension adopted on the phospholipid membrane increased, the limitation on intramolecular motion led to a decreased nonradiative rate constant (which was also understood as a RIM effect), this increases the quantum yield and fluorescence lifetime overall. Compared to the RMSD and RE values of ASP and ASP-OD, those of ASP-PE showed a higher sensitivity to the membrane tension changes, indicating that APE-PE is the best membrane tension probe among the three molecules here, which is in good agreement with the experimental results. Considering that ASP, ASP-PE, and ASP-OD molecules share the same chromophore unit, their photo-inactive moieties led to their most stable positions and postures in the membrane, which, in turn, led to their different sensitivities to the changes of membrane tension.

## DISCUSSION

In summary, with an electron push-pull π-conjugation skeleton and a rarely reported diethyl phosphonate moiety on the pyridinium ring, an AIE-based bioprobe ASP-PE is developed for dual PM-mitochondria–targeting and visualizing cellular mechanical properties. Referring to respective subcellular localization and biomechanical sensitivity of ASP-PE and two structurally similar styryl dyes, the structural comparisons suggest that the phosphonate ester moiety in ASP-PE acts as a mechanosensitivity enhancer in membranous organelles. In addition to sensitivity to different environments during live-cell imaging by FLIM, two distinguishable fluorescence lifetimes are exhibited in the PM and mitochondria, respectively, for ASP-PE.

In high-tension membrane, ASP-PE experiences less freedom of molecular motions and exhibits brighter emission and longer fluorescence lifetime due to the RIM effect. The fluorescence lifetime of ASP-PE in the cell membrane of HeLa cells is linearly correlated with the osmotic pressure in the medium. With the dual targeting feature of ASP-PE, changes of mechanical properties not only in the PM but also in intracellular mitochondria are studied. Different mechanobiological responses in these membrane systems are revealed by the τ_f_ changes of ASP-PE under osmotic shock and varied substrate stiffness. In hypotonic medium or on the high stiffness substrate, the cell membrane is stretched and has a high membrane tension. The probe correspondingly shows a significantly longer fluorescence lifetime. In hypertonic medium or on the low stiffness substrate, the fluorescence lifetimes of ASP-PE in the cell membrane are much shorter. The signal of ASP-PE indicates that the mechanical properties of mitochondria are almost unaffected by substrate stiffness as the PM does. While under osmotic stress, the mitochondria have altered fluorescence lifetime signals with the same trend as the PM. It demonstrates that the probe can be used in studying the relation of the PM and mitochondria.

Furthermore, ASP-PE staining under pharmacological treatments reveals that the PM and mitochondrial membranes are distinctively influenced by microtubules or actin disorganization. This further implies that PM and mitochondrial membranes encounter respective challenges during mechanical stress. Meanwhile, it should be noted that cell contraction or cell volume changes accompanied may alter the focal planes of the PM and mitochondria differently in adherent cells. In those scenarios, it may be challenging to unmix the signals from the PM and mitochondria under the wide-field FLIM. In addition to the experimental data, in silico investigation is used to understand and verify the hypothesis that correlates the membrane tension of PM (or mitochondria) and the fluorescence lifetime of the dual organelle targeting mechanosensitive probe ASP-PE. The computational analysis reveals that the non-π-conjugated units present in the pyridine ring in these three molecules greatly affect their most favorite positions and binding postures in the membrane, which further led to different sensitivities to membrane tension changes.

Together, the promising sensitivity of ASP-PE to mechanical stress and mechanobiological events demonstrates a rapid and reliable approach for exploring cellular mechanosensing. Its potential application in mechanobiological studies can further be widened by modifying this mechanosensitive bioprobe with organelle-targeting signals to reveal organelle mechanosensitivity in real time during dynamic processes, such as morphogenesis and cell migration, with the aid of FLIM.

## MATERIALS AND METHODS

### Materials and reagents

All chemical reactions were performed under an inert nitrogen atmosphere with the use of a Schlenk line. Glassware was dried in an oven before use. Commercially available reagents were used without purification. All the reagents for chemical synthesis were purchased from Tokyo Chemical Industry Co. Ltd., Sigma-Aldrich, Acros Organics, or J&K Scientific. Dry solvents were purchased from the abovementioned companies and stored in the presence of activated 3-Å molecular sieves. All the reactions were monitored by thin-layer chromatography with Merck precoated aluminum plates. Products were purified by column chromatography on silica gel (230 to 400 mesh from Merck or Davisil LC60Å 40 to 63 μm from W. R. Grace & Co.-Conn). The CCK-8 from Yeasen Biotech was used for testing cytotoxicity. The commercially available probes used were BioTracker 405 Blue Mitochondria Dye (Sigma-Aldrich) and CellMask Deep Red Plasma Membrane Stain (Thermo Fisher Scientific). PDMS kits of SYLGARD184 were purchased from Dow Corning. Gelatin, CytoD, and Noc were bought from Sigma-Aldrich.

### Instrumentation for chemical characterizations and photophysical measurements

Proton and carbon NMR spectra were measured in CDCl_3_ on a Bruker AVANCE III 500 (or 400) MHz NMR spectrometer, and tetramethylsilane was exploited as an internal standard for calibrating the chemical shift. Electrospray ionization quadrupole time-of-flight (ESI-Q-TOF) MS was performed on an Agilent 6540 liquid chromatography–ESI-Q-TOF mass spectrometer. UV-Vis absorption spectroscopy was performed on a Molecular Devices SpectraMax M2e in different solutions at 293 K. The solution emission spectra and TCSPC of the chemical probes were measured on a PerkinElmer fluorescence spectrometer LS 55 and FLS1000 PL spectrometer at 293 K, respectively. The fluorescence decay curves were analyzed using an algorithm fitting routine supplied by Fluoracle and were shown to follow a biexponential function.

### Cell culture, staining, and osmotic shock

HeLa cells were cultured in Dulbecco’s modified Eagle’s medium (DMEM; Gibco) supplemented with 10% (v/v) fetal bovine serum (Gibco) and 1% (v/v) penicillin-streptomycin and were maintained in a humidified incubator at 37°C in the presence of 5% CO_2_. Unless specified otherwise, cells were seeded on a gelatin-coated 35-mm glass-bottom cell culture dish (NEST) at least 24 hours before the experiments. The fluorescence staining was performed by incubating the cells with 10 μM ASP-PE, ASP, or ASP-OD at 37°C for 15 min and then washed with phosphate-buffered saline (PBS) for three times before imaging. For the osmotic shock, HeLa cells were stained with the probe, followed by replacing the culture medium with solutions with different osmolarities. Fluorescence lifetime imaging was performed immediately after osmotic shock. Hepes-buffered DMEM (Gibco, 21063029) with ~360 mOsm/liter was treated as isotonic medium. The hypotonic medium (75 and 162 mOsm/liter) were prepared by diluting the Hepes-buffered DMEM with Milli-Q water, whereas the hypertonic medium (482 and 750 mOsm/liter) were prepared by adding NaCl into the Hepes-buffered DMEM.

### Confocal laser scanning microscopy

Confocal imaging was performed using a laser scanning confocal microscope (Zeiss LSM 880) equipped with a Plan-Apochromat 63×/1.40–numerical aperture (NA) objective lens, a photomultiplier tube, and a Gallium arsenide phosphide detector controlled by ZEN software (Carl Zeiss). The excitation lasers and the band-pass filters used were as follows: a 514-nm argon laser and an emission filter of 530 to 630 nm for ASP-PE, ASP, and ASP-OD; a 405-nm diode laser and an emission filter of 410 to 490 nm for BioTracker 405 Blue Mitochondria Dye; and a 633-nm HeNe laser and an emission filter of 655 to 705 nm for CellMask Deep Red Plasma Membrane Stain. Digital images were acquired and pseudo-colored with ZEN software (ZEN 2.5 lite). The degree of colocalization was evaluated by the plug-in JACoP in ImageJ (National Institutes of Health).

### FLIM and analysis

The FLIM core system consisted of a wide-field complementary metal oxide semiconductor frequency-domain FLIM camera (pco.flim, PCO AG, Germany), an inverted fluorescence microscope (Nikon Eclipse Ti2-U, Nikon, Japan) equipped with a Plan-Apochromat Lambda 60×/1.4-NA objective lens, a homogeneously illuminating laser light source (λ_ex_ = 515 nm; pco.flim laser, PCO AG, Germany) with a specified power at 100 mW, and a filter cube from Nikon containing an excitation filter (514/20 nm) and a long-pass emission filter (525 nm). Before the experiment, a reference of a standard sample slide with a known lifetime of 3.55 ns (UMM-SFO 33X, Fluorescent Reference Materials, Starna Scientific, UK) was measured. Throughout all the experiments, the laser power was set to 10% via Omicron Control Center (Omicron-Laserage, Germany) with the exposure time at 100 ms and the modulation frequency at 30 MHz controlled using NIS-Elements AR (Nikon, Japan) integrated with a module for the pco.flim camera. By measuring the phase shift acquired from photons emitted induced by the sinusoidally modulated laser light source, the lifetime map of the interest fields and the lifetime distributions were collected and analyzed using NIS-Elements AR (Nikon, Japan).

### Fabrication of PDMS substrates

The blends of PDMS were prepared using the commercially available SYLGARD 184 (Dow Corning). A blend with 10:1 (v/v, base/curing agent) of SYLGARD 184 PDMS was prepared by stirring. For live-cell imaging, the construction of a PDMS well-included two parts, which were a thin-bottom layer and a frame structure. All the PDMS mixtures were vacuum-degassed in a desiccator for 1 hour. Then, the thin-bottom layer was prepared by the float-on-water method (*37*). Briefly, we used 8 ml of ultrapure water loaded in a 60-mm petri dish as the substratum, dropped an approximately 100 μg of the PDMS blends on it using a 1000-μl pipette tip, and then left it spreading over the surface of the water. Samples were later cured at 75°C for 1 hour in a convection oven. On the other hand, the preparation of the frame structure was achieved by casting the SYLGARD 184 PDMS in a 35-mm petri dish followed by a curing process at 75°C for 1 hour. The inner circular opening (Ø = 5 mm) of the frames was created by a punch. The complete PDMS well was next assembled by putting and pressing the PDMS frame structure onto the thin PDMS layers floated on water for adhesion. The well was coated with 5 μg of type I collagen per centimeter squared at 37°C for at least 4 hours before seeding cells on it.

### Cell viability

The cytotoxicity was evaluated using a standard CCK-8. Briefly, HeLa cells were seeded in a 96-well plate at a density of 7 × 10^3^ cells per well and cultured overnight before treatment. A stock of 10 mM ASP-PE in DMSO was diluted to different concentrations (2.5, 5, 10, 15, or 20 μM) in fresh culture medium, and DMSO was used as the vehicle control. After incubating the cells with the above solutions for 24 hours, 10 μl of CCK-8 stock solution in PBS was added to each well, and cells were incubated for an additional 4 hours. The absorbance was measured using a SpectraMax M2e microplate reader (Molecular Devices) at a wavelength of 450 nm, and the cell viability percentage was calculated from the optical density.

### Tensile testing

The tensile bar strips of the PDMS substrates were prepared by casting the PDMS blends in a mold with shapes in compliance with ASTM D412 Type C and curing at 75°C for 1 hour. Testing was done on Instron 5942 Micro Newton Tester (Instron, Norwood, MA, USA). The samples were stretched at a rate of 50 mm/min until failure, following ASTM D412. The Young’s modulus of the PDMS strips was subsequently determined automatically by the in-house software provided by Instron in which the steepest slope, which is the Young’s modulus, was calculated on the initial linear portion of the curve using least-square fit on the test data.

### Statistical analysis

Statistical significance was determined using two-tailed unpaired *t* test or one-way analysis of variance (ANOVA) with Tukey’s post hoc test. A *P* value less than 0.05 was considered statistically significant, and *n* is the number of independent experiments.

### Computational investigation

Detailed computational methods, including (i) the preparation of ternary component–based phospholipid bilayer model, (ii) MD modeling, (iii) pulling MD simulation, (iv) free-energy profiles simulation, (v) permeability coefficient calculations, and (vi) quantum mechanics/molecular mechanics calculations are fully described in the Supplementary Materials.

## Supplementary Materials

### This PDF file includes:

Supplementary Text Tables S1 and S2 Figs. S1 to S21 References

## References

[1] P. K. Chaudhuri, B. C. Low, C. T. Lim, Mechanobiology of tumor growth. Chem. Rev. 118, 6499–6515 (2018). 29927236 10.1021/acs.chemrev.8b00042

[2] M. Aragona, T. Panciera, A. Manfrin, S. Giulitti, F. Michielin, N. Elvassore, S. Dupont, S. Piccolo, A mechanical checkpoint controls multicellular growth through YAP/TAZ regulation by actin-processing factors. Cell 154, 1047–1059 (2013). 23954413 10.1016/j.cell.2013.07.042

[3] G. R. DiResta, S. S. Nathan, M. W. Manoso, J. Casas-Ganem, C. Wyatt, T. Kubo, P. J. Boland, E. A. Athanasian, J. Miodownik, R. Gorlick, J. H. Healey, Cell proliferation of cultured human cancer cells are affected by the elevated tumor pressures that exist in vivo. Ann. Biomed. Eng. 33, 1270–1280 (2005). 16133932 10.1007/s10439-005-5732-9

[4] J. Das, S. Maji, T. Agarwal, S. Chakraborty, T. K. Maiti, Hemodynamic shear stress induces protective autophagy in HeLa cells through lipid raft-mediated mechanotransduction. Clin. Exp. Metastasis 35, 135–148 (2018). 29536225 10.1007/s10585-018-9887-9

[5] J. Lee, A. A. Abdeen, K. L. Wycislo, T. M. Fan, K. A. Kilian, Interfacial geometry dictates cancer cell tumorigenicity. Nat. Mater. 15, 856–862 (2016). 27043781 10.1038/nmat4610

[6] J. Lee, A. A. Abdeen, Y. Li, S. Goonetilleke, K. A. Kilian, Gradient and dynamic hydrogel materials to probe dynamics in cancer stem cell phenotypes. ACS Appl. Bio Mater. 4, 711–720 (2021).

[7] J. J. Northey, L. Przybyla, V. M. Weaver, Tissue force programs cell fate and tumor aggression. Cancer Discov. 7, 1224–1237 (2017). 29038232 10.1158/2159-8290.CD-16-0733 PMC5679454

[8] A. Diz-Muñoz, D. A. Fletcher, O. D. Weiner, Use the force: Membrane tension as an organizer of cell shape and motility. Trends Cell Biol. 23, 47–53 (2013). 23122885 10.1016/j.tcb.2012.09.006 PMC3558607

[9] A.-L. Le Roux, X. Quiroga, N. Walani, M. Arroyo, P. Roca-Cusachs, The plasma membrane as a mechanochemical transducer. Philos. Trans. R. Soc. B 374, 20180221 (2019). 10.1098/rstb.2018.0221 PMC6627014 31431176

[10] O. Chaudhuri, S. T. Koshy, C. Branco da Cunha, J.-W. Shin, C. S. Verbeke, K. H. Allison, D. J. Mooney, Extracellular matrix stiffness and composition jointly regulate the induction of malignant phenotypes in mammary epithelium. Nat. Mater. 13, 970–978 (2014). 24930031 10.1038/nmat4009

[11] Y. Wang, J. Y.-J. Shyy, S. Chien, Fluorescence proteins, live-cell imaging, and mechanobiology: Seeing is believing. Annu. Rev. Biomed. Eng. 10, 1–38 (2008). 18647110 10.1146/annurev.bioeng.010308.161731

[12] Y.-L. Zhang, J. A. Frangos, M. Chachisvilis, Laurdan fluorescence senses mechanical strain in the lipid bilayer membrane. Biochem. Biophys. Res. Commun. 347, 838–841 (2006). 16857174 10.1016/j.bbrc.2006.06.152

[13] M. A. Boyd, N. P. Kamat, Visualizing tension and growth in model membranes using optical dyes. Biophys. J. 115, 1307–1315 (2018). 30219285 10.1016/j.bpj.2018.08.021 PMC6170595

[14] L. P. Bharath, J. M. Cho, S.-K. Park, T. Ruan, Y. Li, R. Mueller, T. Bean, V. Reese, R. S. Richardson, J. Cai, A. Sargsyan, K. Pires, P. V. Anandh Babu, S. Boudina, T. E. Graham, J. D. Symons, Endothelial cell autophagy maintains shear stress-induced nitric oxide generation via glycolysis-dependent purinergic signaling to eNOS. Arterioscler. Thromb. Vasc. Biol. 37, 1646–1656 (2017). 28684613 10.1161/ATVBAHA.117.309510 PMC5693355

[15] E. E. Mowers, M. N. Sharifi, K. F. Macleod, Functions of autophagy in the tumor microenvironment and cancer metastasis. FEBS J. 285, 1751–1766 (2018). 29356327 10.1111/febs.14388 PMC5992019

[16] R. Bretón-Romero, R. Acín-Perez, F. Rodríguez-Pascual, M. Martínez-Molledo, R. P. Brandes, E. Rial, J. A. Enríquez, S. Lamas, Laminar shear stress regulates mitochondrial dynamics, bioenergetics responses and PRX3 activation in endothelial cells. Biochim Biophys. Acta 1843, 2403–2413 (2014). 25038307 10.1016/j.bbamcr.2014.07.003

[17] C. Ploumi, I. Daskalaki, N. Tavernarakis, Mitochondrial biogenesis and clearance: A balancing act. FEBS J. 284, 183–195 (2017). 27462821 10.1111/febs.13820

[18] A. Colom, E. Derivery, S. Soleimanpour, C. Tomba, M. D. Molin, N. Sakai, M. González-Gaitán, S. Matile, A. Roux, A fluorescent membrane tension probe. Nat. Chem. 10, 1118–1125 (2018). 30150727 10.1038/s41557-018-0127-3 PMC6197433

[19] S. Soleimanpour, A. Colom, E. Derivery, M. Gonzalez-Gaitan, A. Roux, N. Sakai, S. Matile, Headgroup engineering in mechanosensitive membrane probes. Chem. Commun. 52, 14450–14453 (2016). 10.1039/c6cc08771j 27901525

[20] A. Goujon, A. Colom, K. Straková, V. Mercier, D. Mahecic, S. Manley, N. Sakai, A. Roux, S. Matile, Mechanosensitive fluorescent probes to image membrane tension in mitochondria, endoplasmic reticulum, and lysosomes. J. Am. Chem. Soc. 141, 3380–3384 (2019). 30744381 10.1021/jacs.8b13189

[21] K. Straková, J. López-Andarias, N. Jiménez-Rojo, J. E. Chambers, S. J. Marciniak, H. Riezman, N. Sakai, S. Matile, HaloFlippers: A general tool for the fluorescence imaging of precisely localized membrane tension changes in living cells. ACS Cent. Sci. 6, 1376–1385 (2020). 32875078 10.1021/acscentsci.0c00666 PMC7453570

[22] T. C. OwYong, S. Ding, N. Wu, T. Fellowes, S. Chen, J. M. White, W. W. H. Wong, Y. Hong, Optimising molecular rotors to AIE fluorophores for mitochondria uptake and retention. Chem. Commun. 56, 14853–14856 (2020). 10.1039/d0cc06411d 33174870

[23] M.-Y. Wu, A. Y. H. Wong, J.-K. Leung, C. Kam, K. L.-K. Wu, Y.-S. Chan, K. Liu, N. Y. Ip, S. Chen, A near-infrared AIE fluorescent probe for myelin imaging: From sciatic nerve to the optically cleared brain tissue in 3D. Proc. Natl. Acad. Sci. U.S.A. 118, e2106143118 (2021). 34740969 10.1073/pnas.2106143118 PMC8609329

[24] S. Chen, Y. Hong, Y. Zeng, Q. Sun, Y. Liu, E. Zhao, G. Bai, J. Qu, J. Hao, B. Z. Tang, Mapping live cell viscosity with an aggregation-induced emission fluorogen by means of two-photon fluorescence lifetime imaging. Chem. A Eur. J. 21, 4315–4320 (2015). 10.1002/chem.201405658 25645956

[25] J. Mei, N. L. C. Leung, R. T. K. Kwok, J. W. Y. Lam, B. Z. Tang, Aggregation-induced emission: Together we shine, united we soar! Chem. Rev. 115, 11718–11940 (2015). 26492387 10.1021/acs.chemrev.5b00263

[26] L. Magrassi, D. Purves, J. W. Lichtman, Fluorescent probes that stain living nerve terminals. J. Neurosci. 7, 1207–1214 (1987). 3572476 10.1523/JNEUROSCI.07-04-01207.1987 PMC6568996

[27] M. J. McCarthy, J. Baumber, P. H. Kass, S. A. Meyers, Osmotic stress induces oxidative cell damage to rhesus macaque spermatozoa1. Biol. Reprod. 82, 644–651 (2010). 19846599 10.1095/biolreprod.109.080507 PMC2825172

[28] K. Yamamoto, J. Ando, Vascular endothelial cell membranes differentiate between stretch and shear stress through transitions in their lipid phases. Am. J. Physiol. Heart Circ. Physiol. 309, H1178–H1185 (2015). 26297225 10.1152/ajpheart.00241.2015

[29] M. Páez-Pérez, I. López-Duarte, A. Vyšniauskas, N. J. Brooks, M. K. Kuimova, Imaging non-classical mechanical responses of lipid membranes using molecular rotors. Chem. Sci. 12, 2604–2613 (2021). 10.1039/d0sc05874b PMC8179291 34164028

[30] A. Kostic, C. D. Lynch, M. P. Sheetz, Differential matrix rigidity response in breast cancer cell lines correlates with the tissue tropism. PLOS ONE 4, e6361 (2009). 19626122 10.1371/journal.pone.0006361 PMC2709918

[31] S. Li, F. Zhao, Y. Zhan, X. Liu, T. Hun, H. Zhang, C. Qiu, J. He, Z. Yi, Y. Sun, Y. Fan, How deep might myoblasts sense: The effect of substrate stiffness and thickness on the behavior of myoblasts. J. Med. Biol. Eng. 38, 596–606 (2018).

[32] D. E. Discher, P. Janmey, Y.-L. Wang, Tissue cells feel and respond to the stiffness of their substrate. Science 310, 1139–1143 (2005). 16293750 10.1126/science.1116995

[33] M. P. Sheetz, J. E. Sable, H.-G. Döbereiner, Continuous membrane-cytoskeleton adhesion requires continuous accommodation to lipid and cytoskeleton dynamics. Annu. Rev. Biophys. Biomol. Struct. 35, 417–434 (2006). 16689643 10.1146/annurev.biophys.35.040405.102017

[34] J. L. Madara, D. Barenberg, S. Carlson, Effects of cytochalasin D on occluding junctions of intestinal absorptive cells: Further evidence that the cytoskeleton may influence paracellular permeability and junctional charge selectivity. J. Cell Biol. 102, 2125–2136 (1986). 3711143 10.1083/jcb.102.6.2125 PMC2114240

[35] M. Schliwa, Action of cytochalasin D on cytoskeletal networks. J. Cell Biol. 92, 79–91 (1982). 7199055 10.1083/jcb.92.1.79 PMC2112008

[36] A. Grossfield, “*WHAM: The weighted histogram analysis method*,” version 2.0.11; http://membrane.urmc.rochester.edu/wordpress/?page_id=126.

[37] D. Kim, S.-H. Kim, J. Y. Park, Floating-on-water fabrication method for thin polydimethylsiloxane membranes. Polymers 11, 1264 (2019). 31370158 10.3390/polym11081264 PMC6722912

[38] S. Brasselet, F. Cherioux, P. Audebert, J. Zyss, New octupolar star-shaped strucures for quadratic nonlinear optics. Chem. Mater. 11, 1915–1920 (1999).

[39] E. L. Wu, X. Cheng, S. Jo, H. Rui, K. C. Song, E. M. Dávila-Contreras, Y. Qi, J. Lee, V. Monje-Galvan, R. M. Venable, J. B. Klauda, W. Im, CHARMM-GUI membrane builder toward realistic biological membrane simulations. J. Comput. Chem. 35, 1997–2004 (2014). 25130509 10.1002/jcc.23702 PMC4165794

[40] S. Kim, J. Lee, S. Jo, C. L. Brooks III, H. S. Lee, W. Im, CHARMM-GUI ligand reader and modeler for CHARMM force field generation of small molecules. J. Comput. Chem. 38, 1879–1886 (2017). 28497616 10.1002/jcc.24829 PMC5488718

[41] D. A. Case, K. Belfon, I. Y. Ben-Shalom, S. R. Brozell, D. S. Cerutti, T. E. Cheatham III, V. W. D. Cruzeiro, T. A. Darden, R. E. Duke, G. Giambasu, M. K. Gilson, H. Gohlke, A. W. Goetz, R Harris, S. Izadi, S. A. Izmailov, K. Kasavajhala, A. Kovalenko, R. Krasny, T. Kurtzman, T.S. Lee, S. LeGrand, P. Li, C. Lin, J. Liu, T. Luchko, R. Luo, V. Man, K. M. Merz, Y. Miao, O. Mikhailovskii, G. Monard, H. Nguyen, A. Onufriev, F. Pan, S. Pantano, R. Qi, D. R. Roe, A. Roitberg, C. Sagui, S. Schott-Verdugo, J. Shen, C. L. Simmerling, N. R. Skrynnikov, J. Smith, J. Swails, R. C. Walker, J. Wang, L. Wilson, R. M. Wolf, X. Wu, Y. Xiong, Y. Xue, D. M. York, P. A. Kollman, *AMBER 20* (University of California, San Francisco, 2020).

[42] W. L. Jorgensen, J. Chandrasekhar, J. D. Madura, R. W. Impey, M. L. Klein, Comparison of simple potential functions for simulating liquid water. J. Chem. Phys. 79, 926–935 (1983).

[43] C. J. Dickson, R. C. Walker, I. R. Gould, Lipid21: Complex lipid membrane simulations with AMBER. J. Chem. Theory Comput. 18, 1726–1736 (2022). 35113553 10.1021/acs.jctc.1c01217 PMC9007451

[44] W. D. Cornell, P. Cieplak, C. I. Bayly, I. R. Gould, K. M. Merz, D. M. Ferguson, D. C. Spellmeyer, T. Fox, J. W. Caldwell, P. A. Kollman, A second generation force field for the simulation of proteins, nucleic acids, and organic molecules. J. Am. Chem. Soc. 117, 5179–5197 (1995).

[45] V. Hornak, R. Abel, A. Okur, B. Strockbine, A. Roitberg, C. Simmerling, Comparison of multiple Amber force fields and development of improved protein backbone parameters. Proteins 65, 712–725 (2006). 16981200 10.1002/prot.21123 PMC4805110

[46] D. Svozil, J. E. Sponer, I. Marchan, A. Pérez, T. E. Cheatham III, F. Forti, F. J. Luque, M. Orozco, J. Sponer, Geometrical and electronic structure variability of the sugar−phosphate backbone in nucleic acids. J. Phys. Chem. B 112, 8188–8197 (2008). 18558755 10.1021/jp801245h

[47] J. Wang, P. Cieplak, P. A. Kollman, How well does a restrained electrostatic potential (RESP) model perform in calculating conformational energies of organic and biological molecules? J. Comput. Chem. 21, 1049–1074 (2000).

[48] Gaussian 16, Revision B.01, M. J. Frisch, G. W. Trucks, H.B. Schlegel, G. E. Scuseria, M. A. Robb, J. R. Cheeseman, G. Scalmani, V. Barone, G. A. Petersson, H. Nakatsuji, X. Li, M. Caricato, A. V. Marenich, J. Bloino, B. G. Janesko, R. Gomperts, B. Mennucci, H. P. Hratchian, Ortiz, J. V., A. F. Izmaylov, J. L. Sonnenberg, D. Williams-Young, F. Ding, F. Lipparini, F. Egidi, J. Goings, B. Peng, A. Petrone, T. Henderson, D. Ranasinghe, V. G. Zakrzewski, J. Gao, N. Rega, G. Zheng, W. Liang, M. Hada, M. Ehara, K. Toyota, R. Fukuda, J. Hasegawa, M. Ishida, T. Nakajima, Y. Honda, O. Kitao, H. Nakai, T. Vreven, K. Throssell, J. A. Montgomery Jr., J. E. Peralta, F. Ogliaro, M. J. Bearpark, J. J. Heyd, E. N. Brothers, K. N. Kudin, V. N. Staroverov, T. A. Keith, R. Kobayashi, J. Normand, K. Raghavachari, A. P. Rendell, J. C. Burant, S. S. Iyengar, J. Tomasi, M. Cossi, J. M. Millam, M. Klene, C. Adamo, R. Cammi, J. W. Ochterski, R. L. Martin, K. Morokuma, O. Farkas, J. B. Foresman, D. J. Fox, *GaussView 5.0.* (Gaussian Inc., 2016).

[49] X. Wu, B. R. Brooks, Self-guided Langevin dynamics simulation method. Chem. Phys. Lett. 381, 512–518 (2003).

[50] J.-P. Ryckaert, G. Ciccotti, H. J. C. Berendsen, Numerical integration of the cartesian equations of motion of a system with constraints: Molecular dynamics of *n*-alkanes. J. Comput. Phys. 23, 327–341 (1977).

[51] G. M. Torrie, J. P. Valleau, Nonphysical sampling distributions in Monte Carlo free-energy estimation: Umbrella sampling. J. Comput. Phys. 23, 187–199 (1977).

[52] R. Vijayaraj, S. Van Damme, P. Bultinck, V. Subramanian, Molecular dynamics and umbrella sampling study of stabilizing factors in cyclic peptide-based nanotubes. J. Phys. Chem. B 116, 9922–9933 (2012). 22804626 10.1021/jp303418a

[53] S. Kumar, J. M. Rosenberg, D. Bouzida, R. H. Swendsen, P. A. Kollman, THE weighted histogram analysis method for free-energy calculations on biomolecules. I. The method. J. Comput. Chem. 13, 1011–1021 (1992).

[54] Extension to the weighted histogram analysis method: Combining umbrella sampling with free energy calculations. Comput. Phys. Commun. 135, 40–57 (2001).

[55] C. T. Lee, J. Comer, C. Herndon, N. Leung, A. Pavlova, R. V. Swift, C. Tung, C. N. Rowley, R. E. Amaro, C. Chipot, Y. Wang, J. C. Gumbart, Simulation-based approaches for determining membrane permeability of small compounds. J. Chem. Inf. Model. 56, 721–733 (2016). 27043429 10.1021/acs.jcim.6b00022 PMC5280572

[56] G. Hummer, Position-dependent diffusion coefficients and free energies from Bayesian analysis of equilibrium and replica molecular dynamics simulations. New J. Phys. 7, 34–34 (2005).

[57] G. Scalmani, M. J. Frisch, Continuous surface charge polarizable continuum models of solvation. I. General formalism. J. Chem. Phys. 132, 114110 (2010). 20331284 10.1063/1.3359469

[58] S. Dapprich, I. Komáromi, K. S. Byun, K. Morokuma, M. J. Frisch, A new ONIOM implementation in Gaussian98. Part I. The calculation of energies, gradients, vibrational frequencies and electric field derivatives. J. Mol. Struct. Theochem 461, 1–21 (1999).

[59] O. A. Vydrov, G. E. Scuseria, Assessment of a long-range corrected hybrid functional. J. Chem. Phys. 125, 234109 (2006). 17190549 10.1063/1.2409292

[60] L. A. Curtiss, M. P. McGrath, J. P. Blaudeau, N. E. Davis, R. C. Binning Jr., L. Radom, Extension of Gaussian-2 theory to molecules containing third-row atoms Ga–Kr. J. Chem. Phys. 103, 6104–6113 (1995).

[61] J. Andzelm, E. Wimmer, Density functional Gaussian-type-orbital approach to molecular geometries, vibrations, and reaction energies. J. Chem. Phys. 96, 1280–1303 (1992).

[62] M. Petersilka, U. J. Gossmann, E. K. U. Gross, Excitation energies from time-dependent density-functional theory. Phys. Rev. Lett. 76, 1212–1215 (1996). 10061664 10.1103/PhysRevLett.76.1212

[63] R. C. Hilborn, Einstein coefficients, cross sections,f values, dipole moments, and all that. Am. J. Phys. 50, 982–986 (1982).

[64] Y. Niu, W. Li, Q. Peng, H. Geng, Y. Yi, L. Wang, G. Nan, D. Wang, Z. Shuai, MOlecular MAterials Property Prediction Package (MOMAP) 1.0: A software package for predicting the luminescent properties and mobility of organic functional materials. Mol. Phys. 116, 1078–1090 (2018).

[65] Q. Peng, Y. Yi, Z. Shuai, J. Shao, Toward quantitative prediction of molecular fluorescence quantum efficiency: Role of Duschinsky rotation. J. Am. Chem. Soc. 129, 9333–9339 (2007). 17622142 10.1021/ja067946e

[66] Z. Shuai, Q. Peng, Excited states structure and processes: Understanding organic light-emitting diodes at the molecular level. Phys. Rep. 537, 123–156 (2014).

[67] Z. Shuai, Q. Peng, Organic light-emitting diodes: Theoretical understanding of highly efficient materials and development of computational methodology. Natl. Sci. Rev. 4, 224–239 (2017).

[68] S. Braslavsky, Glossary of terms used in photochemistry 3rd edition (IUPAC Recommendations 2006). Pure Appl. Chem. 79, 293–465 (2007).

