# Supplementary material for: A dual organelle-targeting mechanosensitive probe
Source: Sci Adv. 2023 Jan 11;9(2):eabn5390. doi: 10.1126/sciadv.abn5390 (PMC9833668; doi:10.1126/sciadv.abn5390)
Supplement: Supplementary file 1 — Supplementary Text Tables S1 and S2 Figs. S1 to S21 References [file sciadv.abn5390_sm.pdf]

Science Advances
AAAS

Supplementary Materials for

A dual organelle-targeting mechanosensitive probe

Po-Yu Ho *et al.*

Corresponding author: Sijie Chen, sijie.chen@ki.se

Sci. Adv. **9**, eabn5390 (2023)
DOI: 10.1126/sciadv.abn5390

This PDF file includes:

Supplementary Text
Tables S1 and S2
Figs. S1 to S21
References

Supplementary Text

Synthetic procedures of chemical compounds

The intermediate, (*E*)-*N*,*N*-diethyl-4-(2-(pyridin-4-yl)vinyl)aniline (i.e., negative staining control), was first synthesized by a modified published procedure (*38*). A mixture of 4-diethylaminobenzaldehyde (2 g, 11.28 mmol), 4-methylpyridine (1.05 g, 11.28 mmol) and sodium *tert*-butoxide (1.302 g, 13.546 mmol) in anhydrous dimethylformamide (20 mL) was heated to 80 °C with stirring under nitrogen atmosphere for 2 hours. The reaction mixture, after cooling down, was poured into water (100 mL). Vigorous stirring was kept, and an orange slurry was formed. The orange participate was filtered out and washed with water and minimum amount of ethanol and diethyl either. The solid reside was purified by multiple recrystallization of (i) toluene and then (ii) dichloromethane:hexane mixture to afford intermediate (*E*)-*N*,*N*-diethyl-4-(2-(pyridin-4-yl)vinyl)aniline (655 mg, 2.594 mmol, 23%) as shiny yellow crystals. ^{1}H NMR ($CDCl_3$, 500 MHz): δ = 8.50 (dd, 2H, J = 5, 1.5 Hz, Ar), 7.41 (d, 2H, J = 9 Hz, Ar), 7.30 (dd, 2H, J = 5, 1.5 Hz, Ar), 7.22 (d, 1H, J = 16.5 Hz, C=CH), 6.76 (d, 1H, J = 16 Hz, C=CH), 6.66 (d, 2H, J = 8.5 Hz, Ar), 3.40 (q, 4H, J = 7 Hz, alkyl), 1.19 ppm (t, 6H, J = 7 Hz, alkyl); ^{13}C NMR (125 MHz, $CDCl_3$): δ = 150.09, 148.29, 145.82, 133.52, 128.72, 123.36, 120.60, 120.45, 111.59 (Ar), 44.55, 12.75 ppm (alkyl). HRMS (MALDI-TOF, *m*/*z*): [$(M+H)^+$] 253.1705; calcd for ($C_{17}H_{21}N_2^+$) 253.1699.

ASP-PE was next synthetized as follows: a mixture of (*E*)-*N*,*N*-diethyl-4-(2-(pyridin-4-yl)vinyl)aniline (150 mg, 0.595 mmol) and diethyl(3-bromopropyl)phosphonate (169 mg, 0.652 mmol) in acetonitrile (4 mL) was heated to 85 °C with stirring under nitrogen atmosphere overnight. After cooling, the solvent was removed under reduced pressure. The residue was purified by column chromatography on silica gel using a 1:10 mixture of CH_3OH and methylene chloride as eluent to give ASP-PE (230 mg, 0.450 mmol, 76%) as a sticky deep red oil. ^{1}H NMR ($CDCl_3$, 400 MHz): δ = 9.11 (d, 2H, J = 6.8 Hz, Ar), 7.73 (d, 2H, J = 6.8 Hz, Ar), 7.58 (d, 1H, J = 16 Hz, C=CH), 7.50 (d, 2H, J = 9.2 Hz, Ar), 6.81 (d, 1H, J = 16 Hz, C=CH), 6.69 (d, 2H, J = 9.2 Hz, Ar), 4.92 (t, 2H, J = 7.2 Hz, alkyl), 4.15−4.07 (m, 4H, alkyl), 3.46−3.42 (m, 4H, alkyl), 2.46−2.36 (m, 2H, alkyl), 1.92−1.84 (m, 2H, alkyl), 1.34 (t, 6H, J = 7.2 Hz, alkyl), 1.23 ppm (t, 6H, J = 7.2 Hz, alkyl); ^{13}C NMR (125 MHz, $CDCl_3$): δ = 154.51, 150.30, 143.59, 143.34, 131.19, 122.50, 121.67, 115.75, 111.60 (Ar), 62.25, 62.20, 59.05, 58.94, 58.35, 58.32, 44.73, 25.10, 25.07, 22.12, 20.99, 18.50, 16.54, 16.50, 12.68 ppm (alkyl). HRMS (MALDI-TOF, *m*/*z*): [$(M-Br)^+$] 431.2460; calcd for ($C_{24}H_{36}N_2O_3P^+$) 431.2458.

ASP-OD was synthetized as follows: a mixture of (*E*)-*N*,*N*-diethyl-4-(2-(pyridin-4-yl)vinyl)aniline (100 mg, 0.396 mmol) and 9-(bromomethyl)nonadecane (160 mg, 0.443 mmol) in acetonitrile (4 mL) was heated to 85 °C with stirring under nitrogen atmosphere overnight. After cooling, the solvent was removed under reduced pressure. The residue was purified by column chromatography on silica gel using a 1:10 mixture of CH_3OH and methylene chloride as eluent to give ASP-OD (63 mg, 0.103 mmol, 26%) as a sticky deep red oil. ^{1}H NMR ($CDCl_3$, 500 MHz): δ = 8.74 (d, 2H, J = 6.5 Hz, Ar), 7.84 (d, 2H, J = 6.5 Hz, Ar), 7.63 (d, 1H, J = 16 Hz, C=CH), 7.51 (d, 2H, J = 9 Hz, Ar), 6.83 (d, 1H, J = 16 Hz, C=CH), 6.68 (d, 2H, J = 8.5 Hz, Ar), 4.47 (d, 2H, J = 7.5 Hz, alkyl), 3.44 (q, 4H, J = 7 Hz, alkyl), 1.98−1.94 (m, 1H, alkyl), 1.36−1.21 (m, 38H, alkyl), 0.87 ppm (t, 6H, J = 7 Hz, alkyl); ^{13}C NMR (125 MHz, $CDCl_3$): δ = 154.38, 143.59, 143.34, 131.23, 122.59, 121.84, 115.86, 111.66 (Ar), 64.24, 60.45, 44.81, 40.29, 32.03, 31.96, 30.62, 29.92, 29.73, 29.60, 29.55, 29.46, 29.38, 26.23, 22.81, 22.78, 14.25, 12.74 ppm (alkyl). HRMS (MALDI-TOF, *m*/*z*): [$(M-Br)^+$] 533.4838; calcd for ($C_{37}H_{61}N_2^+$) 533.4829.

Detailed computational method

1.1 Preparation of simulation systems

Firstly, the Bilayer Builder Module (*39*) on the Charmm-GUI website (https://charmm-gui.org/) was selected to build the phospholipid bilayer. During the construction process, the 1-palmitoyl-2-oleoyl-sn-glycero-3-phosphocholine (POPC) phospholipids, cholesterol (CHOL), and *N*-palmitoyl-d-erythro-sphingosylphosphorylcholine (PSM) phospholipids were selected as the components of the phospholipid bilayer, and the numbers of them in the phospholipid bilayer were 120, 40, and 40, respectively. In other words, there were in totally 200 phospholipids in our constructed phospholipid bilayer. Meanwhile, the water layer containing 0.15 mol/L neutralizing ions (KCl) was also added upon the two phospholipid layers of the phospholipid bilayer. In the final constructed pure phospholipid bilayer model, the thickness of both water layers upon the phospholipid layer were 35 Å (a total of 14,543 water molecules and 38 KCl molecules). Secondly, about 50 ns molecular dynamics simulation was employed for the constructed pure phospholipid bilayer model to obtain an equilibrium state of the pure phospholipid bilayer. Detailed molecular dynamics (MD) simulation processes were described below. Finally, the Ligand Reader & Modeler Module (*40*) on the Charmm-GUI website was further selected to build the 3D structures of ASP, ASP-PE and ASP-OD molecules, and then, along the Z-axis they were placed into the center of the pure phospholipid bilayer that has reached an equilibrium state, respectively. The placement processes of the molecules into the center of phospholipid bilayers were realized by a written python script. Thus, besides one pure phospholipid bilayer model, three phospholipid bilayer models were also constructed with their corresponding center positions occupied by the ASP, ASP-PE and ASP-OD molecules oriented along the Z-axis, respectively.

1.2 Molecular dynamics simulations

For above four constructed phospholipid bilayer models, the Leap program integrated in the Amber20 package (*41*) were firstly employed to add hydrogen atoms for the POPC phospholipids, CHOL phospholipids, PSM phospholipids, ASP molecules, ASP-PE molecules, ASP-OD molecules, and water molecules, and then, the tools integrated in the AmberTools package (*41*) were employed to solvate every model with hydrogen added in a rectangular box described by the TIP3P water model (*42*). The length, width and height of the rectangular box was determined by that of every model with hydrogen added (the lengths, widths and heights of the four phospholipid bilayer models were measured in the VMD package, and all four models were 79.803997 Å × 79.014999 Å × 121.046001 Å). Before the molecular dynamics simulations, the force field parameters should also been added for the phospholipids, ASP molecules, ASP-PE molecules, ASP-OD molecules and water molecules. Herein, in the four models, the newly reported Lipid21 force field (*43*) was applied to describe the behaviors of the POPC phospholipids, CHOL phospholipids and PSM phospholipids. The FF14SB force field (*44-46*) which was provided by Amber20 package (*41*) was applied to describe the behaviors of the TIP3P model, and the General Amber Force Field 2 (GAFF2) (*47*) which was also provided by Amber20 package (*41*) was applied to describe the behaviors of the ASP, ASP-PE and ASP-OD molecules. Meanwhile, the partial atomic charges of the ASP, ASP-PE and ASP-OD molecules were obtained from the restrained electrostatic potential (RESP) charges firstly computed at the HF/6-31G* level with the Gaussian16 package (*48*) and then restricted fitted with the antechamber program in Amber20 (*41*).

The MD simulations of the phospholipid bilayer models with water box and neutralizing ions added were performed in Amber20 by employing GPU-accelerated pmemd.cuda module (*41*).

Every model prepared above was firstly optimized by three steps: (i) 2000 cycles of steepest descent and 2000 cycles of conjugate gradient were carried out to relax the solvent (water molecules and neutralizing ions), while the entire phospholipid bilayer as well as the ASP, ASP-PE and ASP-OD molecules were constrained by a potential of 2000 kcal/mol·Å^2. (ii) Another 2000 cycles of steepest descent and 2000 cycles of conjugate gradient were executed to minimize the entire model with the heavy atoms of the phospholipid bilayer, ASP molecules, ASP-PE molecules and ASP-OD molecules constrained by 500 kcal/mol·Å^2. (iii) Further 2000 cycles of steepest descent and 2000 cycles of conjugate were employed to minimize the entire model only with the heavy atoms of the ASP, ASP-PE and ASP-OD molecules constrained by 200 kcal/mol·Å^2. Subsequently, every optimized model was gradually heated from 0 to 300 K under the NVT ensemble for 200 ps, followed by another 200 ps of molecular dynamics simulations to relax the system density to about 1.0 g/cm^3 at 300 K under the NPT ensemble. Finally, with a target temperature of 300 K, about 50 ns NPT molecular dynamics simulations were further performed to equilibrate every phospholipid bilayer model. In order to ensure that the ASP, ASP-PE and ASP-OD molecules were always in the center of the phospholipid bilayer models during the molecular dynamics processes, the positions of the heavy atoms of the ASP, ASP-PE and ASP-OD molecules were constrained by 50 kcal/mol·Å^2 during the heating stage, the density adjustment stage and the final equilibrium stage. During the entire molecular dynamics simulations, a cutoff of 12 Å was set for both van der Waals and electrostatic interactions, and the Langevin dynamics method (*49*) was used to control the system temperature with a collision frequency of 1.0 ps^{-1} (ntt = 3, gamma_ln = 1.0). Meanwhile, the SHAKE algorithm (*50*) was applied to constrain all of the hydrogen-containing bonds with a tolerance of 10^{-5}.

<u>1.3 Pulling molecular dynamics simulations</u>

The three models with their corresponding center positions occupied by the ASP, ASP-PE and ASP-OD molecules oriented along the Z-axis after the 50 ns molecular dynamics simulations were further chosen as the initial structures to study the change processes of the ASP, ASP-PE and ASP-OD molecules moving from the center of the phospholipid bilayer to the water phase. And the change processes were performed in Amber20 by employing GPU-accelerated pmemd.cuda module (*41*) to run the pulling molecular dynamics simulations. During the pulling molecular dynamics processes, the Z-axis component of the center-of-mass (COM) distance between the center of phospholipid bilayer and each molecule of the ASP, ASP-PE and ASP-OD molecules were chosen as the pulling coordinate for each model, and each molecule of the ASP, ASP-PE and ASP-OD molecules was pulled from the center of the phospholipid bilayer (Z = 0 Å) out to the water phase (Z = 35 Å) at a pulling rate of 1 Å/ns and a restraint force constant of 2.5 kcal/mol·Å^2 by running 35 ns molecular dynamics simulation under NPT ensemble with semi-isotropic. The restraint force applied on the ASP, ASP-PE and ASP-OD molecules was only along the Z-axis, while along the X-axis and Y-axis they were allowed to move freely. Meanwhile, during 35 ns pulling molecular dynamics simulations, the configurations were recorded with a time interval of 2 ps and in total 17,500 configurations along the pulling coordinate (i.e., Z-axis) from 0 Å to 35 Å were collected for each model.

<u>1.4 Potential of mean force simulations</u>

To determine the free energy profiles of translocation of the ASP, ASP-PE and ASP-OD molecules from the center of the phospholipid bilayer to the water phase, the umbrella sampling techniques (*51, 52*) which could determine the potential of mean force (i.e., free energy) of translocation of

the three molecules were used. For each model, 36 determined configurations along the pulling coordinate (i.e., Z-axis) from 0 Å to 35 Å with an interval of 1 Å from the pulling molecular dynamics simulation were selected as the initial structures to perform the molecular dynamics simulations with umbrella sampling. Therefore, in total 36 windows along the Z-axis were setup for each model. For each window, 100 ns molecular dynamics simulation with a restraint force constant of 2.5 kcal/mol·Å^2 applied on the corresponding pulling coordinate was performed to ensure sampling of the entire system of each model realized. During the 100 ns bias molecular dynamics simulation processes, the initial 20 ns was used for equilibration, and following 80 ns were used for sampling of the pulling coordinate data. For each model, the sampled pulling coordinate data for every window were collected and analyzed by the Weighted Histogram Analysis Method (WHAM) (*53, 54*) to map out the free energy profiles of translocation of the corresponding molecule. And the produced free energy profiles (i.e., potential of mean force profiles) for the ASP, ASP-PE and ASP-OD molecules were finally shifted to ensure that the free energy data in water phase were equal to 0 since the free energy profiles were relative. All above potential of mean force simulations were also performed in Amber20 by employing GPU-accelerated pmemd.cuda module (*41*).

1.5 Permeability coefficients calculations

Firstly, besides the free energy profiles, the sampled pulling coordinate data for every window were also used to calculated the Z-dependent diffusion of the three molecules of ASP, ASP-PE and ASP-OD by using the following formula (*55*):

$$D(z) = \frac{var(z)^2}{\int_0^\infty C_{zz}(t)\,dt}$$

Where D(z) was the Z-dependent diffusion, var(z) was the variance of the sampled pulling coordinate data of the window at Z-position, and $C_{zz}(t)$ was the autocorrelation function of the sampled pulling coordinate data of the window at Z-position, which was calculated by the following formula (*55*):

$$C_{zz}(t) = \frac{1}{N}\sum_{i=0}^{N} \delta_z(i)\delta_z(t+i)$$

Where *N* was the sample number of the sampled pulling coordinated data of the window at Z-position, and $\delta_z(i) = z(i) - \langle z \rangle$, where z(i) was the ith sampled pulling coordinated data and $\langle z \rangle$ was the average value of the sampled pulling coordinated data.

Secondly, the obtained Z-dependent diffusion and the free energy data at each Z-position relative to that in water phase (Z = 35 Å) were used to get the Z-dependent resistance of the three molecules of ASP, ASP-PE and ASP-OD by using the following formula (*56*):

$$R(z) = \frac{exp\left(\beta \Delta G(z)\right)}{D(z)}$$

Where the *R*(z) was the Z-dependent resistance, *ΔG*(z) was the free energy data at each Z-position relative to that in water phase (Z = 35 Å), *D*(z) was the Z-dependent diffusion, and $\beta = 1/(k_BT)$, where k_B was the Boltzmann constant (1.38064852×10^{-23} J/K) and T was the simulation temperature (300 K).

Thirdly, the obtained the Z-dependent resistance data were used to integrate over the Z-position, which produced the overall effective resistance coefficients of the three molecules of ASP, ASP-PE and ASP-OD. And the inverse of the overall effective resistance coefficients justly was the overall effective permeability coefficients of the three molecules of ASP, ASP-PE and

ASP-OD. The following formula was used to calculated the overall effective resistance coefficient and the overall effective permeability coefficient (*56*):

$$P_{eff} = \frac{1}{R_{eff}} = \frac{1}{\int_{z_{min}}^{z_{max}} R(z)\,dz}$$

Where the R_{eff} was the overall effective resistance coefficient, P_{eff} was the overall effective permeability coefficient, and R(z) was the Z-dependent resistance.

1.6 Molecular properties calculations

In order to understand the effect of phospholipid membrane on the fluorescence properties of the ASP, ASP-PE and ASP-OD molecules as well as the fluorescence properties differences among the three molecules, the excitation properties of ASP, ASP-PE and ASP-OD molecules in pure ethanol solvent and in phospholipid membranes with different surface tensions (+50, 0 and −200 dyn/cm, 0 dyn/cm meant no surface tension) were calculated by the quantum mechanics (QM) method with polarizable continuum model (PCM) (*57*) and the quantum mechanics/molecular mechanics (QM/MM) method with ONION model (*58*), respectively. Before the calculations of excited states, for each molecule of ASP, ASP-PE and ASP-OD, three conformations were firstly randomly extracted from above obtained umbrella sampling structures at the window with the largest partition (windows at Z = 18 Å, Z = 11 Å and Z = 0 Å for ASP, ASP-PE and ASP-OD molecules, respectively). And then, the three extracted conformations were employed to proceed with another 100 ns molecular dynamics simulations to sample under the conditions that a surface tension of +50 dyn/cm and a surface tension of −200 dyn/cm was applied on the phospholipid membranes, respectively. Finally, additional three conformations were further randomly extracted from the sampling trajectory for every surface tension. Therefore, for the three molecules of ASP, ASP-PE and ASP-OD, a total of 27 conformations were extracted under the three kinds of surface tensions (+50, 0, −200 dyn/cm).

All above obtained 27 conformations were employed to process the QM/MM calculations to obtain the electron excitation properties. Firstly, the water molecules and neutralizing ions beyond 40 Å away from the ASP, ASP-PE and ASP-OD molecules were removed. And then, the ASP, ASP-PE and ASP-OD molecules were selected as the QM region and treated by the LC-ωHPBE method (*59*) with the 6-31+G(d, p) basis set (*60*), while the remaining phospholipid membranes, water molecules and neutralizing ions were selected as the MM region and treated by the Lipid21 force field (*43*) and the FF14SB force field (*44-46*). Finally, in the case that the MM region was frozen, the density functional theory (DFT) (*61*) was used to process the geometry optimizations of the ground state (S_0 state) for the QM regions of the 27 conformations, and based on the optimized conformations of the QM regions, the time-dependent DFT (TD-DFT) (*62*) was used to process the excited state calculations which could provide the electron excitation properties of absorption spectrums and the optimizations of the first singlet excited state (S_1 state) which could provide the electron excitation properties of emission spectrums. In addition, the electron excitation properties in the pure ethanol solvent were also calculated by the DFT and TD-DFT at the LC-ωHPBE/6-31+G(d, p) level (*59-62*) for the ASP, ASP-PE and ASP-OD molecules. The Gaussian16 package (*48*) was employed to complete all the QM/MM calculations for the ASP, ASP-PE and ASP-OD molecules in the phospholipid membranes and the QM calculations for the ASP, ASP-PE and ASP-OD molecules in the ethanol solvent.

The four-point method were employed to calculate the reorganization energies of S_0 states (λ(I) reorganization energy) and S_1 states (λ(II) reorganization energy). Firstly, the energies of the four points were determined, and they were the electronic energy of the optimized S_0 state (E1),

the electronic energy of S_1 state which was calculated based on the optimized structure of S_0 state (E2), the electronic energy of S_0 state which was calculated based on the optimized structure of S_1 state (E3), and the electronic energy of the optimized S_1 state, respectively. All the four points energies could be directly obtained from above geometry optimizations results of the S_0 state and S_1 state. The λ(I) and λ(II) reorganization energies were justly equal to (E3 − E1) and (E2 – E4), respectively. And for each molecule of ASP, ASP-PE and ASP-OD, no matter in phospholipid membrane or in pure ethanol solvent, its total reorganization energy (RE) was considered as the sum of λ(I) and λ(II) reorganization energies, i.e., RE = E3 + E2 − E1 − E4. The RE was calculated for all the 27 QM/MM systems in the phospholipid membranes and 3 QM systems in the pure ethanol solvent.

Based on the calculated electron excitation properties of emission spectrum which provided the oscillator strength (*f*) and the excitation energy (*v*) between the S_0 state and S_1 state at the optimized geometry structure of S_1 state, the radiative rate constant (k_r) could be obtained via the following formula (*63*):

$$k_r = \frac{v^2 * f}{1.499194}$$

where the units of k_r and v were time (unit: s^{-1}) and wavenumber (unit: cm^{-1}, could be converted from ev unit: cm^{-1} = 8065.5447 × ev), respectively. The k_r was calculated for all the 27 QM/MM systems in the phospholipid membranes and 3 QM systems in the pure ethanol solvent.
Based on the optimized structures of S_0 and S_1 states, the frequency calculation and non-adiabatic coupling calculation were further processed also by employing the Gaussian16 package (*11*), and the outputted vibrational modes and non-adiabatic coupling results were used to obtain the non-radiative rate constant (k_{IC}, also called internal conversion rate constant) by employing the MOMAP 2022A package (*64-67*) which adopted the following formula (*67*) to calculate the k_{IC}.

$$k_{IC} = \frac{2\pi}{\hbar} \sum_{v_i, v_f} \left\{ P_{iv_i}(T) \left| \sum_k \langle \Phi_f | \hat{P}_k | \Phi_i \rangle \langle \Theta_{fv_f} | \hat{P}_k | \Theta_{iv_i} \rangle \right|^2 \delta(E_{iv_i} - E_{fv_f}) \right\}$$

where *i* and *f* were the initial S_1 state and the final S_0 state, respectively. And Φ_i and Φ_j were the wavefunctions of the electron motions for the initial S_1 state and the final S_0 state, respectively. Meanwhile, v_i and v_f represented the vibrational modes in the initial S_1 state and the final S_0 state, respectively. And Θiv_i and Θfv_f represented the wavefunctions of the vibrational mode of v_i in the initial S_1 state and the vibrational mode of v_f in the final S_0 state, respectively. $\hat{P}_k$ was the nuclear momentum operator for the kth vibrational mode. In addition, $\hbar$ was the reduced Planck constant and was equal to $h/2\pi$ (h was the Planck constant and was equal to 6.62606876×10^{-34} J·s). $Piv_i(T)$ was the statistical averaged weight of the vibrational mode of v_i in the initial state. Moreover, Eiv_i and Efv_f reflected the vibrational energies of the vibrational mode of v_i in the initial S_1 state and the vibrational mode of v_f in the final S_0 state, respectively. And their energy difference was dealt with the Dirac delta (δ) function. The k_{IC} was calculated only for the ASP-PE molecules in the phospholipid membranes with different surface tensions (+50, 0 and −200 dyn/cm) and the ASP-PE molecule in the pure ethanol solvent.

Based on above obtained radiative rate constant (k_r) and non-radiative rate constant (k_{IC}), the quantum yield (φ) and the fluorescence lifetime (τ) were respectively calculated via the following formulas (*68*):

$$\varphi = \frac{k_r}{k_r + k_{IC}} \qquad \tau = \frac{1}{k_r + k_{IC}}$$

it should be noted that the intersystem cross rate constant (k_{ISC}) which also should be included in the non-radiative rate constant was not considered in our calculations since its effect in our systems was negligible.

Table S1. Prediction of membrane permeability of ASP, ASP-PE, and ASP-OD.
Calculated effective resistance coefficients and effective permeability coefficients of ASP, ASP-PE and ASP-OD molecules in the phospholipid membrane.

/	ASP	ASP-PE	ASP-OD
**effective resistance coefficient (s/cm)**	3.212739×10^{6}	7.476734	0.028329
**effective permeability coefficient (cm/s)**	3.112609×10^{-7}	0.133748	35.299479

Table S2. Calculated photophysical properties of ASP, ASP-PE, and ASP-OD.
Calculated absorption data, emission data, transition orbital assignments of the optimized S_1 states, radiative rate constant, non-radiative rate constant and fluorescence lifetime.

Models	Absorption (nm)	f_{abs}	Emission (nm)	f_{em}	Assignment of S_1	Radiative rate (s^{-1})	Non-radiative rate (s^{-1})	fluorescence lifetime (ns)
in Ethanol solution								
**ASP**	367.38	1.4630	604.76	1.8279	HOMO-1→LUMO (2.53%)<br>HOMO→LUMO (93.05%)	3.33×10^8	/	/
**ASP-PE**	369.50	1.5486	606.26	1.8657	HOMO-1→LUMO (2.50%)<br>HOMO→LUMO (93.07%)	3.40×10^8	1.22×10^{11}	0.01
**ASP-OD**	367.11	1.5644	593.53	1.8497	HOMO-1→LUMO (2.35%)<br>HOMO→LUMO (93.12%)	3.49×10^8	/	/
ASP in phospholipid bilayer membrane (surface tension = +50 dyn/cm)								
**Frame 1**	419.69	1.4276	509.55	1.4656	HOMO-1→LUMO (5.70%)<br>HOMO→LUMO (87.72%)<br>HOMO→LUMO+2 (3.54%)	3.76×10^8	/	/
**Frame 2**	421.94	1.4999	508.05	1.4850	HOMO-1→LUMO (5.69%)<br>HOMO→LUMO (88.01%)<br>HOMO→LUMO+2 (3.07%)	3.83×10^8	/	/
**Frame 3**	422.37	1.4450	507.94	1.4262	HOMO-1→LUMO (5.42%)<br>HOMO→LUMO (88.45%)<br>HOMO→LUMO+2 (2.80%)	3.69×10^8	/	/
ASP-PE in phospholipid bilayer membrane (surface tension = +50 dyn/cm)								
**Frame 1**	406.06	1.4187	500.15	1.6707	HOMO-1→LUMO (5.09%)<br>HOMO→LUMO (88.91%)<br>HOMO→LUMO+2 (2.53%)	4.44×10^8	5.63×10^9	0.16
**Frame 2**	406.65	1.3769	499.31	1.5812	HOMO-1→LUMO (6.00%)<br>HOMO→LUMO (88.24%)<br>HOMO→LUMO+2 (2.29%)	4.24×10^8	/	/
**Frame 3**	408.02	1.3577	497.85	1.6032	HOMO-1→LUMO (5.33%)<br>HOMO→LUMO (88.96%)<br>HOMO→LUMO+2 (2.39%)	4.31×10^8	/	/
ASP-OD in phospholipid bilayer membrane (surface tension = +50 dyn/cm)								
**Frame 1**	410.47	1.4336	517.08	1.4827	HOMO-1→LUMO (5.25%)<br>HOMO→LUMO (89.04%)<br>HOMO→LUMO+2 (2.05%)	3.70×10^8	/	/
**Frame 2**	411.66	1.4463	515.35	1.5394	HOMO-1→LUMO (5.81%)<br>HOMO→LUMO (88.68%)<br>HOMO→LUMO+2 (2.07%)	3.86×10^8	/	/
**Frame 3**	412.07	1.4414	514.53	1.5046	HOMO-1→LUMO (5.62%)<br>HOMO→LUMO (88.43%)<br>HOMO→LUMO+2 (2.35%)	3.79×10^8	/	/
ASP in phospholipid bilayer membrane (surface tension = 0 dyn/cm)								
**Frame 1**	426.13	1.3935	503.17	1.4688	HOMO-1→LUMO (5.65%)<br>HOMO→LUMO (88.12%)<br>HOMO→LUMO+2 (3.27%)	3.88×10^8	/	/
**Frame 2**	426.99	1.5053	503.42	1.5231	HOMO-1→LUMO (5.23%)<br>HOMO→LUMO (89.06%)<br>HOMO→LUMO+2 (2.79%)	4.11×10^8	/	/
**Frame 3**	428.65	1.4371	502.38	1.5104	HOMO-1→LUMO (5.32%)<br>HOMO→LUMO (88.60%)<br>HOMO→LUMO+2 (2.99%)	3.98×10^8	/	/
ASP-PE in phospholipid bilayer membrane (surface tension = 0 dyn/cm)								
**Frame 1**	410.86	1.5151	482.30	1.5679	HOMO-1→LUMO (6.20%)<br>HOMO→LUMO (87.16%)<br>HOMO→LUMO+2 (3.08%)	4.50×10^8	2.56×10^9	0.33
**Frame 2**	413.84	1.5871	484.60	1.6255	HOMO-1→LUMO (5.35%)<br>HOMO→LUMO (88.70%)<br>HOMO→LUMO+2 (2.63%)	4.61×10^8	/	/
**Frame 3**	411.04	1.5067	483.75	1.6052	HOMO-1→LUMO (5.34%)<br>HOMO→LUMO (88.72%)<br>HOMO→LUMO+2 (2.63%)	4.57×10^8	/	/
ASP-OD in phospholipid bilayer membrane (surface tension = 0 dyn/cm)								
**Frame 1**	417.80	1.4663	503.87	1.5744	HOMO-1→LUMO (5.26%)<br>HOMO→LUMO (88.57%)<br>HOMO→LUMO+2 (2.53%)	4.13×10^8	/	/
**Frame 2**	418.06	1.5588	502.61	1.5596	HOMO-1→LUMO (6.28%)<br>HOMO→LUMO (87.68%)<br>HOMO→LUMO+2 (2.66%)	4.12×10^8	/	/
**Frame 3**	419.14	1.4362	501.59	1.5358	HOMO-1→LUMO (5.37%)<br>HOMO→LUMO (88.90%)<br>HOMO→LUMO+2 (2.25%)	4.07×10^8	/	/
ASP in phospholipid bilayer membrane (surface tension = −200 dyn/cm)								

**Frame 1**	431.79	1.3630	498.06	1.5250	HOMO-1→LUMO (5.69%)<br>HOMO→LUMO (88.01%)<br>HOMO→LUMO+2 (3.07%)	4.10×10^{8}	/	/
**Frame 2**	428.77	1.4942	499.08	1.5486	HOMO-1→LUMO (5.12%)<br>HOMO→LUMO (89.12%)<br>HOMO→LUMO+2 (2.70%)	4.15×10^{8}	/	/
**Frame 3**	432.88	1.4165	497.25	1.5654	HOMO-1→LUMO (5.71%)<br>HOMO→LUMO (87.71%)<br>HOMO→LUMO+2 (3.54%)	4.22×10^{8}	/	/
ASP-PE in phospholipid bilayer membrane (surface tension = −200 dyn/cm)								
**Frame 1**	413.68	1.5122	449.55	1.4913	HOMO-1→LUMO (6.02%)<br>HOMO→LUMO (88.18%)<br>HOMO→LUMO+2 (2.39%)	4.93×10^{8}	1.07×10^{9}	0.64
**Frame 2**	412.85	1.5036	453.52	1.5911	HOMO-1→LUMO (5.88%)<br>HOMO→LUMO (88.08%)<br>HOMO→LUMO+2 (2.82%)	5.15×10^{8}	/	/
**Frame 3**	412.06	1.4933	457.20	1.5265	HOMO-1→LUMO (5.44%)<br>HOMO→LUMO (88.74%)<br>HOMO→LUMO+2 (2.52%)	4.88×10^{8}	/	/
ASP-OD in phospholipid bilayer membrane (surface tension = −200 dyn/cm)								
**Frame 1**	425.24	1.5263	499.08	1.6749	HOMO-1→LUMO (5.48%)<br>HOMO→LUMO (88.34%)<br>HOMO→LUMO+2 (2.48%)	4.48×10^{8}	/	/
**Frame 2**	425.11	1.4126	500.99	1.6892	HOMO-1→LUMO (5.96%)<br>HOMO→LUMO (87.32%)<br>HOMO→LUMO+2 (3.44%)	4.49×10^{8}	/	/
**Frame 3**	423.65	1.5355	501.36	1.6573	HOMO-1→LUMO (5.93%)<br>HOMO→LUMO (88.45%)<br>HOMO→LUMO+2 (2.18%)	4.41×10^{8}	/	/

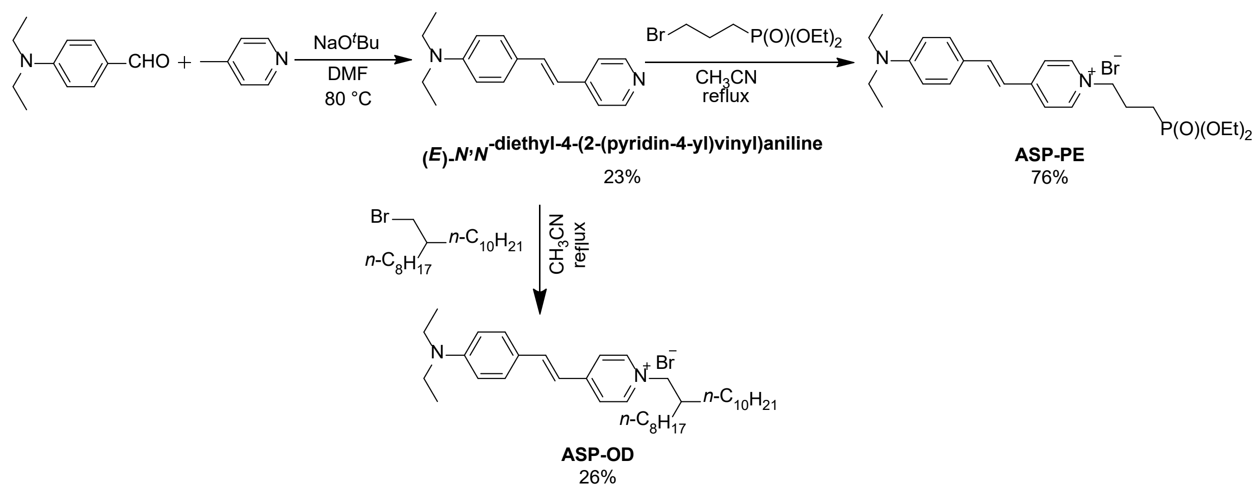

Fig. S1. Synthetic route for (*E*)-*N*,*N*-diethyl-4-(2-(pyridin-4-yl)vinyl)aniline, ASP-PE and ASP-OD. DMF = dimethylformamide.

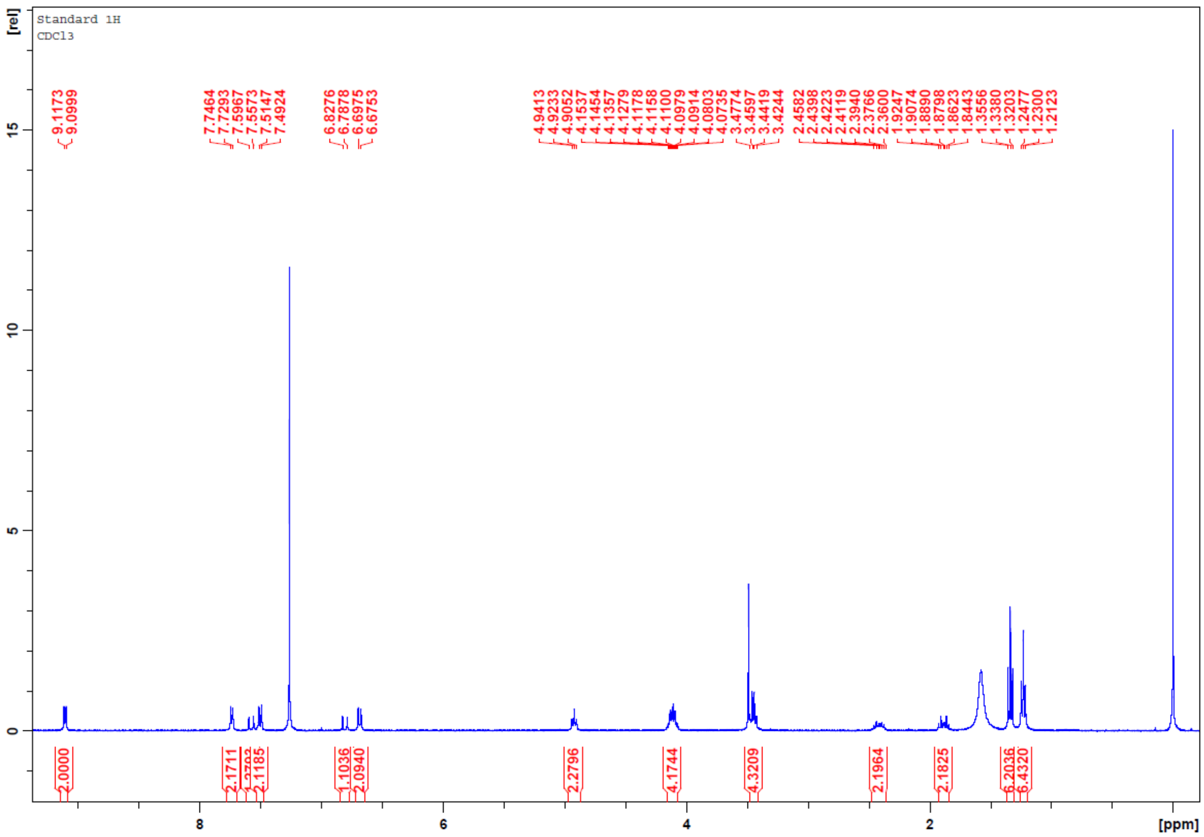

Fig. S2. ^{1}H NMR of ASP-PE in $CDCl_3$.

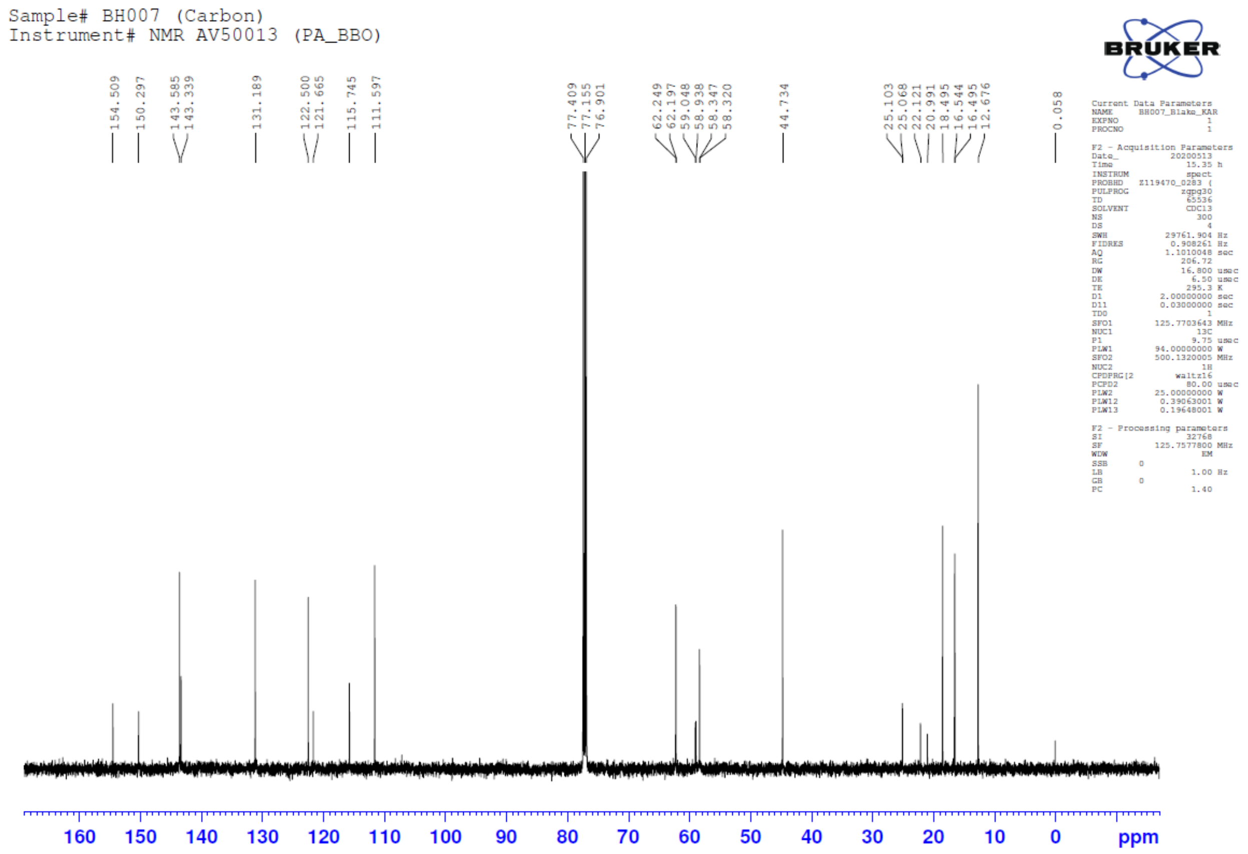

Fig. S3. ^{13}C NMR of ASP-PE in $CDCl_3$.

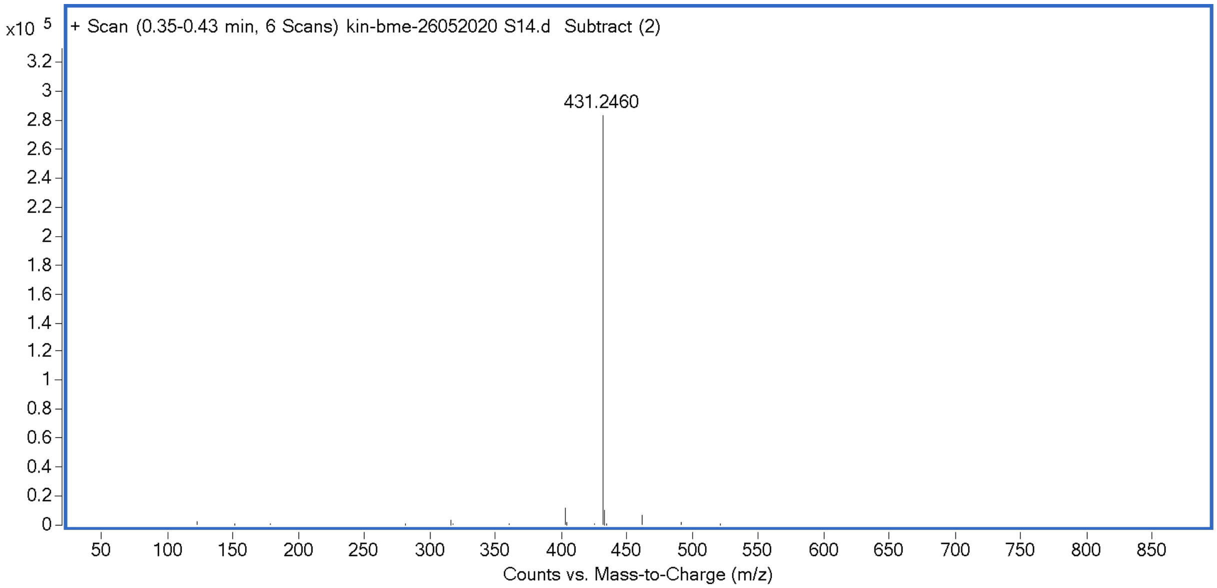

Fig. S4. High resolution ESI-MS spectrum of ASP-PE.

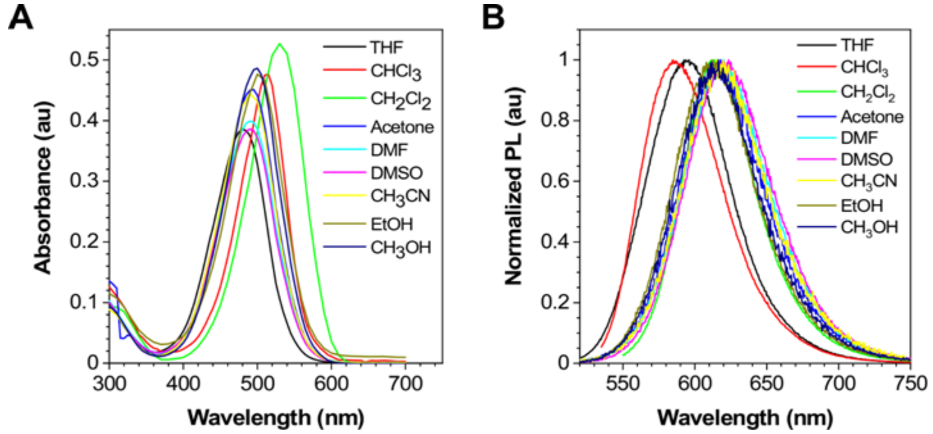

Fig. S5. Absorption and emission spectra of ASP-PE.
(**A**) UV-Vis absorption spectra and (**B**) normalized photoluminescence spectra of ASP-PE (10 μM) in different organic solvents at 293K.

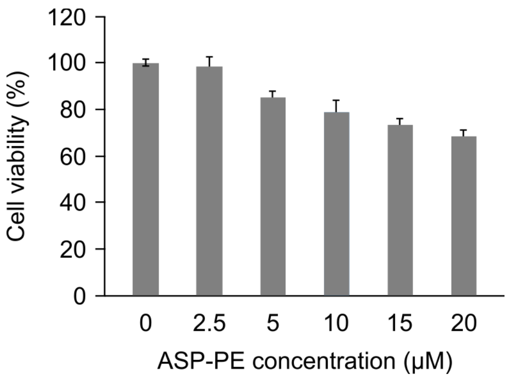

Fig. S6. Cell viability assay of ASP-PE.
Cell viability of HeLa cells incubated with different concentrations of ASP-PE was determined by the CCK-8 assay. Data represent mean ± s.d., *n* = three independent experiments.

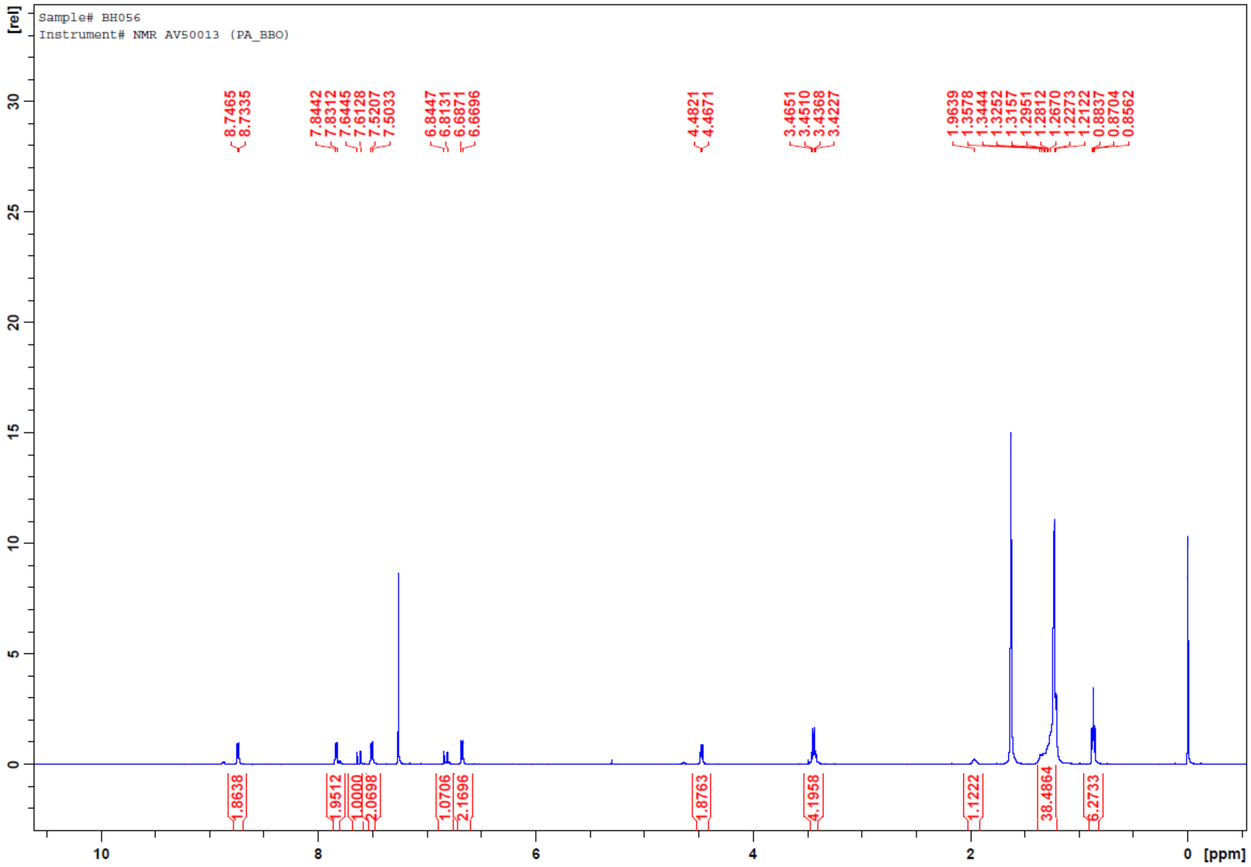

Fig. S7. **^{1}H NMR of ASP-OD in $CDCl_3$.**

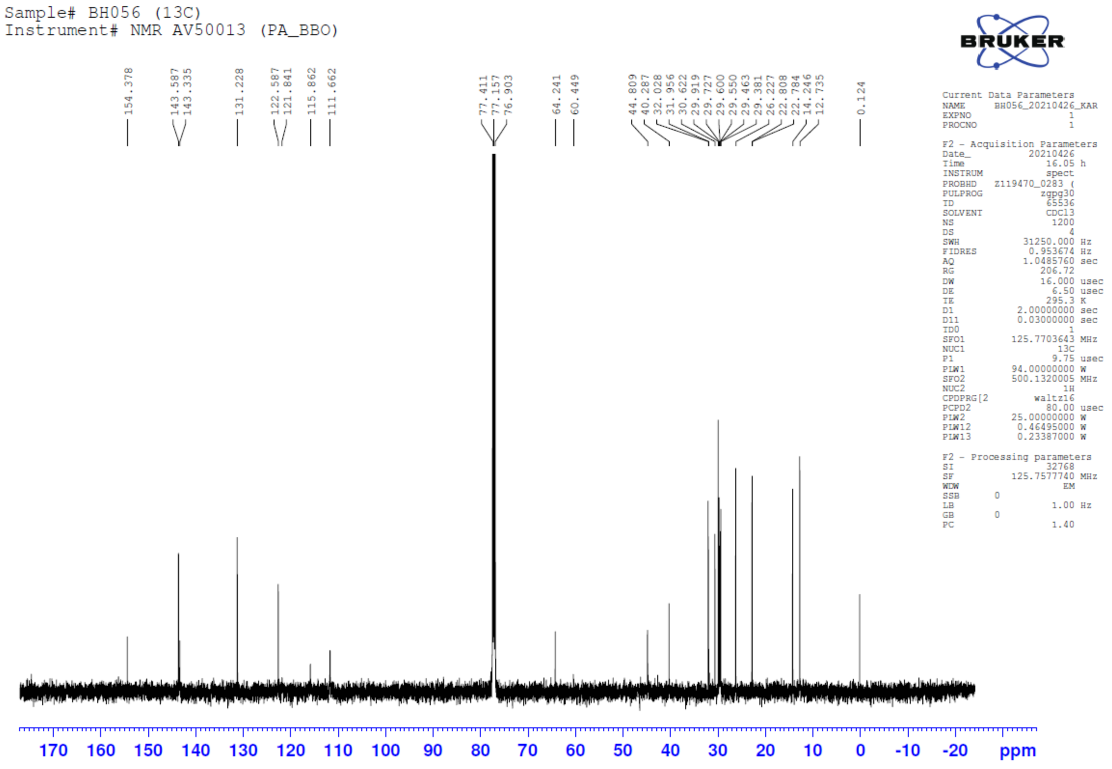

Fig. S8. ^{13}C NMR of ASP-OD in $CDCl_3$.

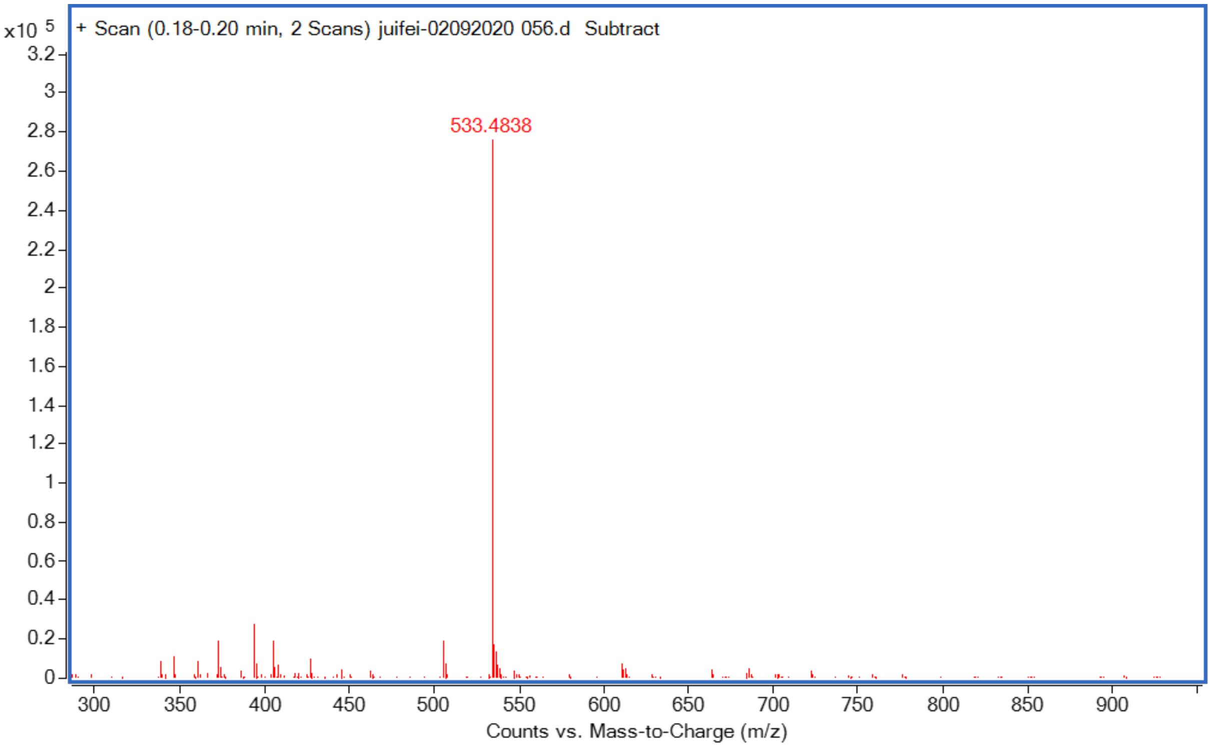

Fig. S9. High resolution ESI-MS spectrum of ASP-OD.

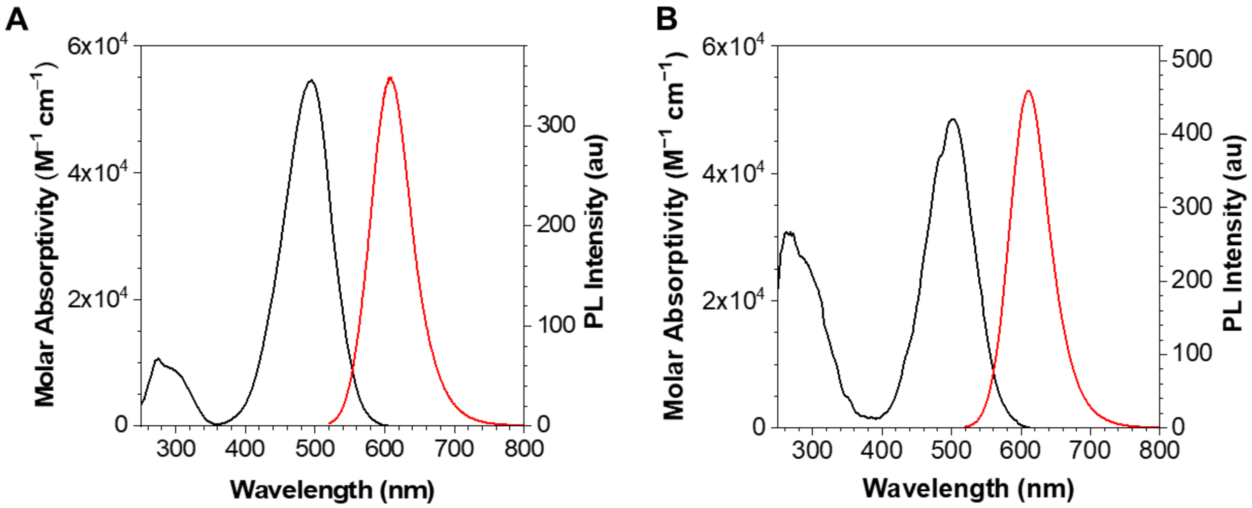

Fig. S10. Absorption and emission spectra of ASP and ASP-OD.
UV-Vis absorption spectra (black lines) and PL spectra (red lines) of (**A**) ASP (10 μM) and (**B**) ASP-OD (10 μM) in ethanol at 293K.

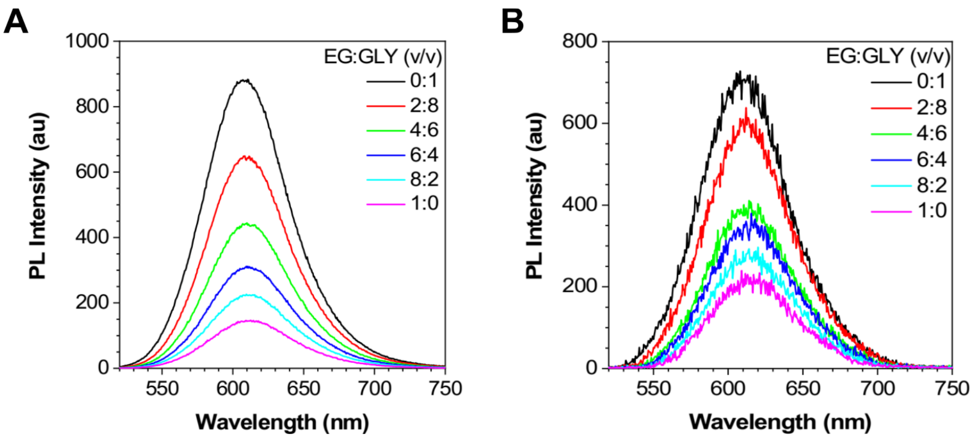

Fig. S11. Photoluminescence spectra of ASP and ASP-OD.
Photoluminescence spectra of (**A**) ASP (10 μM) and (**B**) ASP-OD (10 μM) in different volume ratios of ethylene glycol (EG) and glycerol (GLY) at 293K.

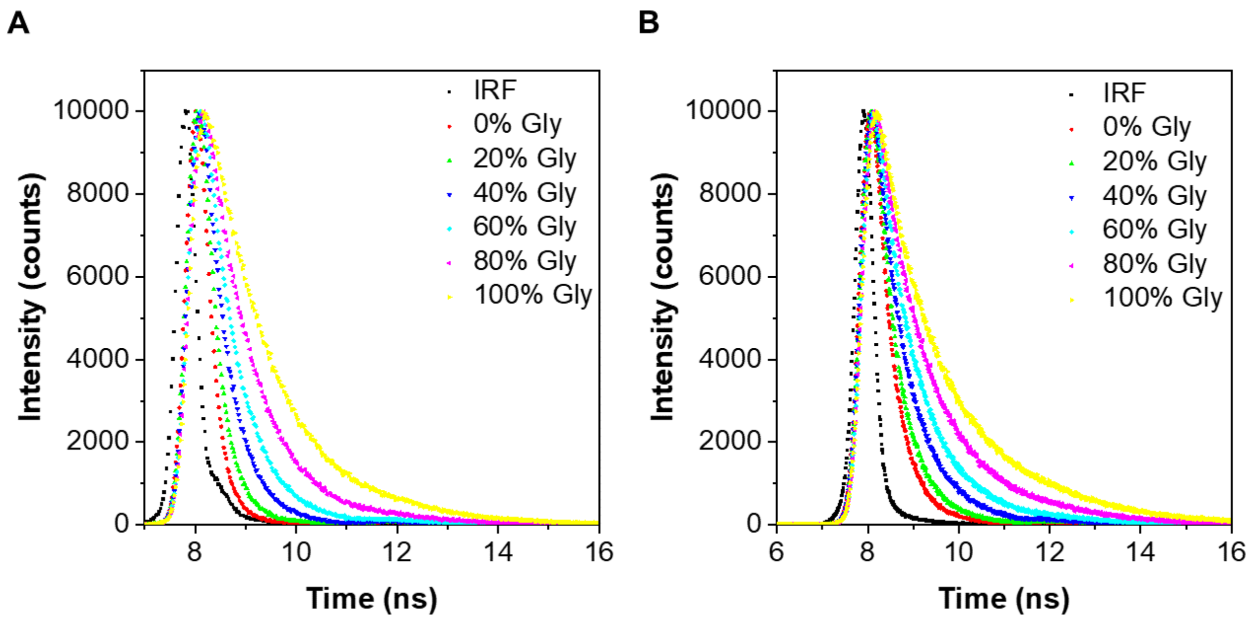

Fig. S12. TCSPC spectra of ASP and ASP-OD.
TCSPC spectra of (**A**) ASP (10 μM) and (**B**) ASP-OD (10 μM) in different volume ratios of ethylene glycol and glycerol (GLY) at 293K. The fitting algorithm analysis calculate the fluorescence lifetime(s) of 0/20/40/60/80/100% Gly solution mixtures. They are reported to be 0.23, 0.32, 0.48, 0.66, 0.94 and 1.29 ns, respectively, for ASP and 0.44, 0.56, 0.75, 1.00, 1.32 and 1.69 ns, respectively, for ASP-OD. IRF, instrumental response function.

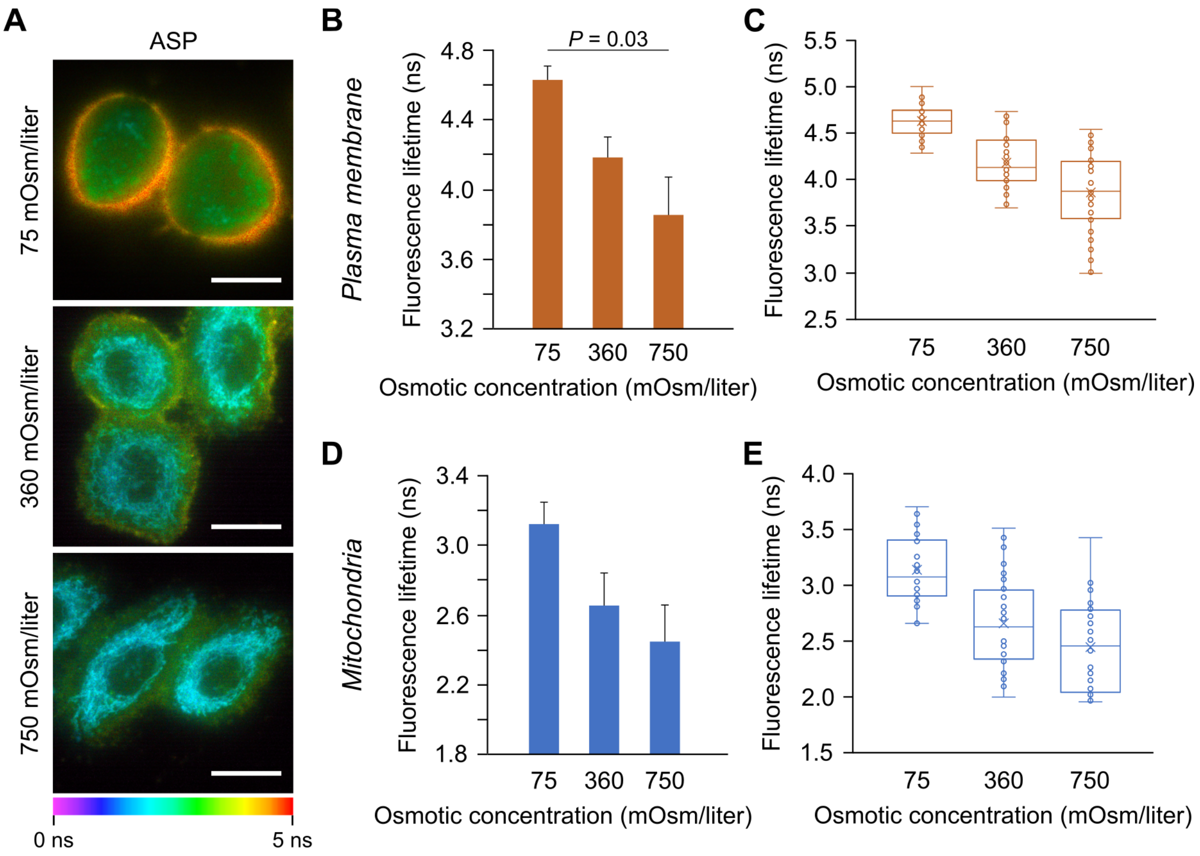

Fig. S13. Response of ASP lifetime in HeLa cells upon osmotic stress.
(**A**) Fluorescence lifetime images of ASP in HeLa cells treated with hypotonic medium (75 mOsm/liter), isotonic medium (360 mOsm/liter), or hypertonic medium (750 mOsm/liter). Scale bars: 20 µm. (**B** to **E**) Quantification of ASP fluorescence lifetime in the plasma membrane (B, C) and mitochondria (D, E). Data represent mean ± s.e.m., n = four independent experiments. Exact P values are shown. Statistical differences were determined by a one-way ANOVA with Tukey's post hoc test (B, D). Box and whisker plot display fluorescence lifetime of ASP in individual cells (C, E).

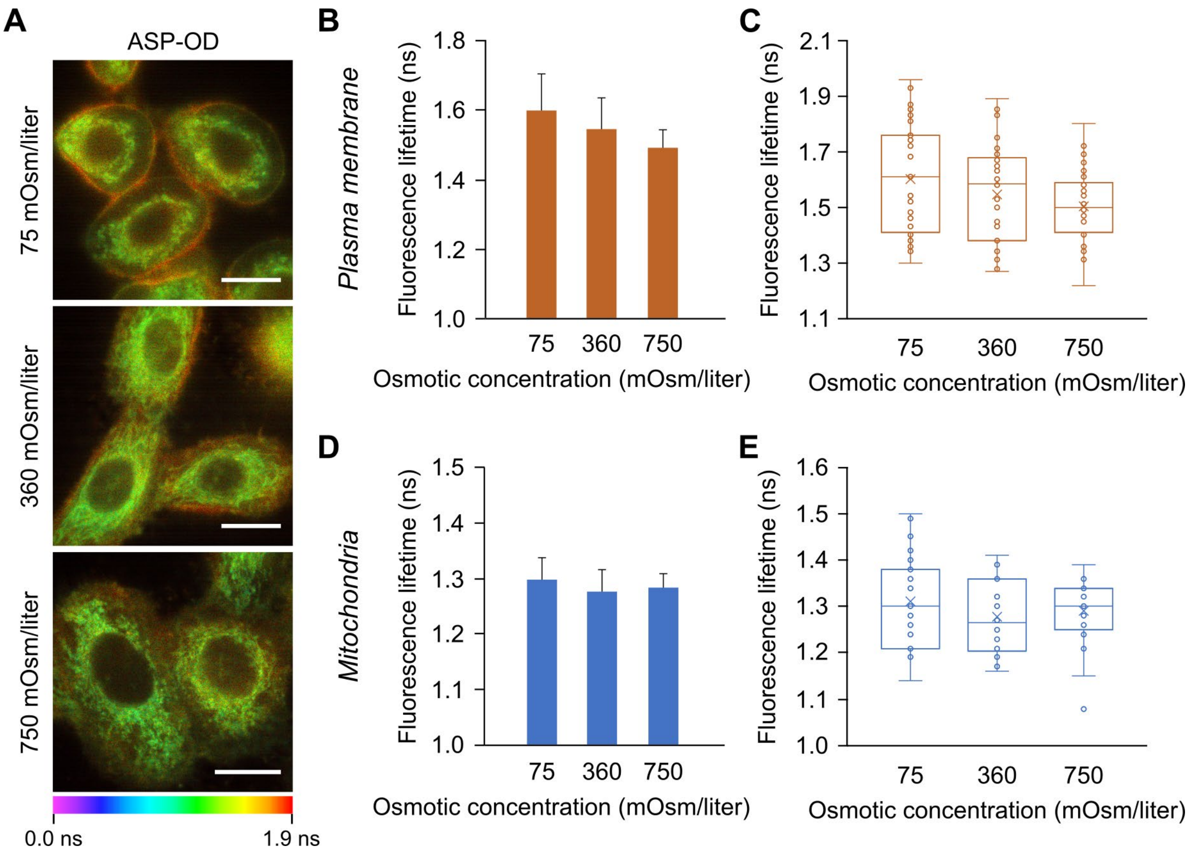

Fig. S14. Response of ASP-OD lifetime in HeLa cells upon osmotic stress.
(**A**) Fluorescence lifetime images of ASP-OD in HeLa cells treated with hypotonic medium (75 mOsm/liter), isotonic medium (360 mOsm/liter), or hypertonic medium (750 mOsm/liter). Scale bars: 20 µm. (**B** to **E**) Quantification of ASP-OD fluorescence lifetime in the plasma membrane (B, C) and mitochondria (D, E). Data represent mean ± s.e.m., n = four independent experiments (B, D). Box and whisker plot display fluorescence lifetime of ASP-OD in individual cells (C, E).

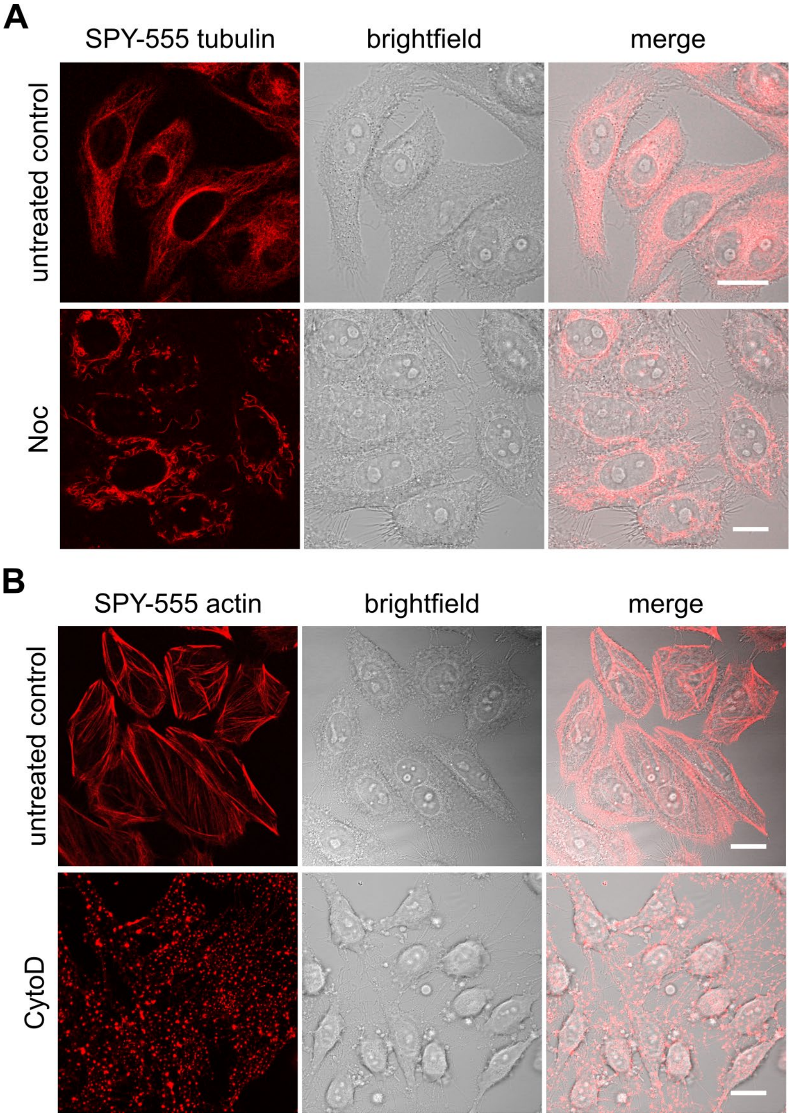

Fig. S15. Pharmacological treatments in HeLa cells.
(**A**) Representative images of HeLa cells with no treatment or treated with 17 μM Noc for 15 min. Tubulin is stained with SPY555-tubulin. Scale bars: 20 μm. (**B**) Representative images of HeLa cells with no treatment or treated with 2 μM CytoD for 30 min. Actin is stained with SPY555-actin. Scale bars: 20 μm.

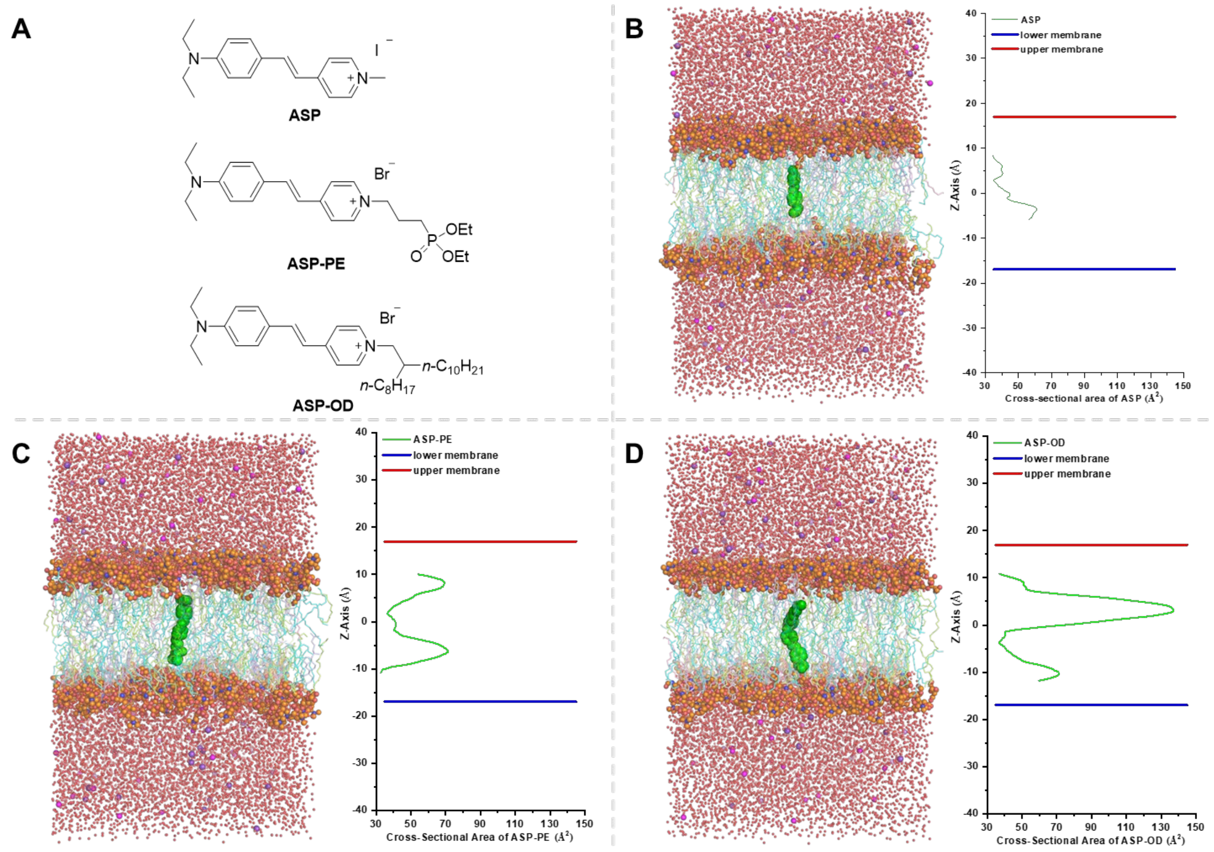

Fig. S16. ASP, ASP-PE and ASP-OD molecules in phospholipid membrane models.
(**A**) Chemical structures of ASP, ASP-PE and ASP-OD molecules. (**B** to **D**) Three constructed phospholipid bilayer models with their phospholipid membrane center occupied by the ASP, ASP-PE and ASP-OD molecules, respectively, as well as the cross-section areas of the three molecules in phospholipid bilayer. The phosphatidylcholine head groups of POPC phospholipids and the sphingomyelin head groups of PSM phospholipids were shown as orange spheres. The palmitoyl acyl chains and oleoyl acyl chains of POPC phospholipids were shown as cyan sticks and limon sticks, respectively. The palmitoyl acyl chains and sphingosine acyl chains of PSM phospholipids were shown as cyan sticks and pink sticks, respectively. The CHOL phospholipids were shown as light blue sticks. The ASP, ASP-PE and ASP-OD molecules were shown as green spheres. Meanwhile, the neutralizing K ions and Cl ions were shown as purple spheres and magenta spheres. The water molecules were shown as red spheres. The POPC, CHOL and PSM phospholipids constituted the components of phospholipid bilayer.

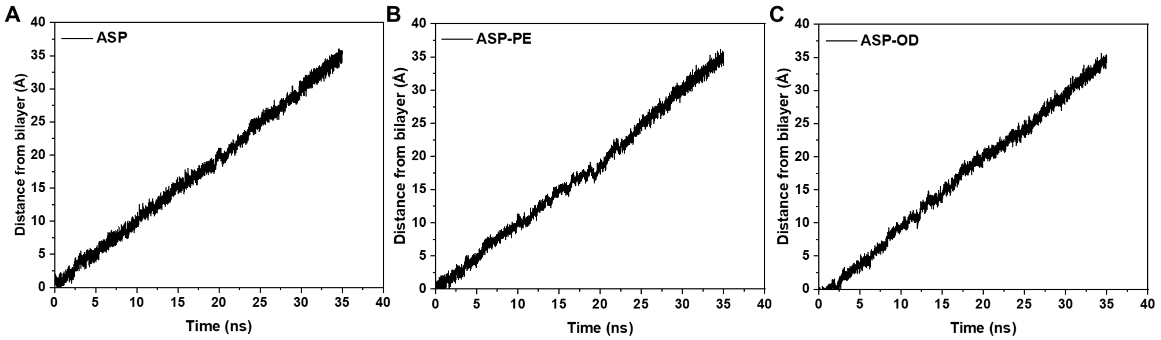

Fig. S17. The pulling molecular dynamics simulations.
The changes of the centroid distances between the ASP (A), ASP-PE (B), and ASP-OD (C) molecules and the phospholipid membrane center along with the simulation time during the pulling molecular dynamics simulations.

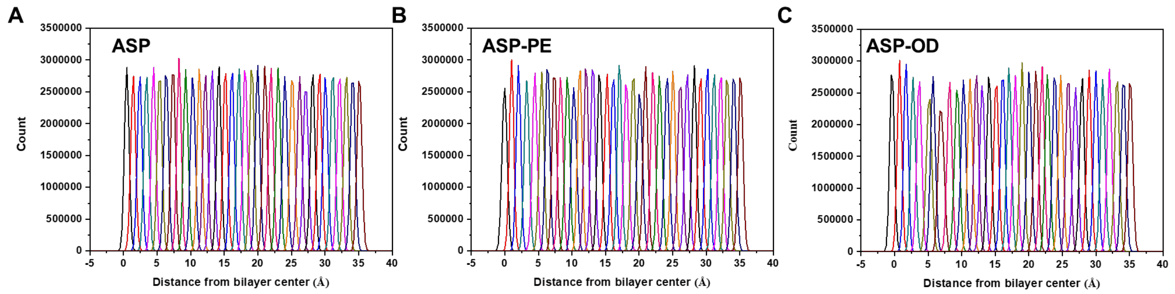

Fig. S18. The window histograms of umbrella sampling.
Overlap of the histogram distributions of the centroid distances between the ASP (A), ASP-PE (B), and ASP-OD (C) molecules and the phospholipid membrane center among different windows.

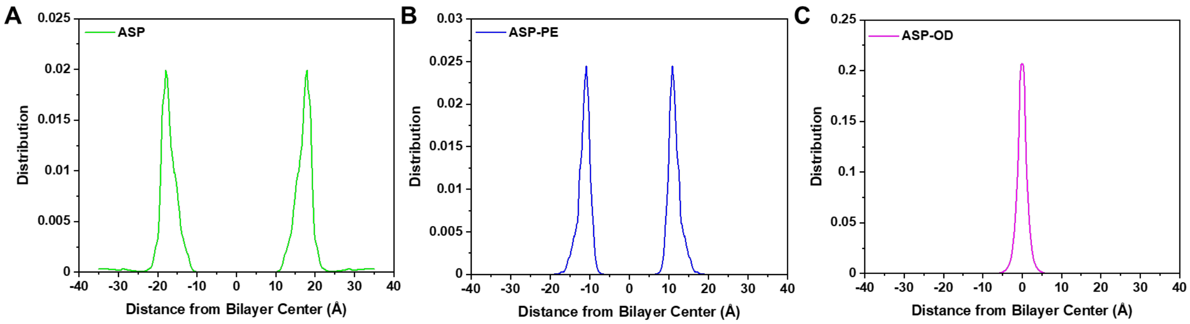

Fig. S19. The distribution probability of ASP, ASP-PE and ASP-OD molecules in phospholipid bilayers.

Different positions of ASP (A), ASP-PE (B), and ASP-OD (C) molecules from the center of the phospholipid membrane were obtained from the potential of mean force profiles. The phospholipid membrane center served as the reference of Z-position as 0 Å.

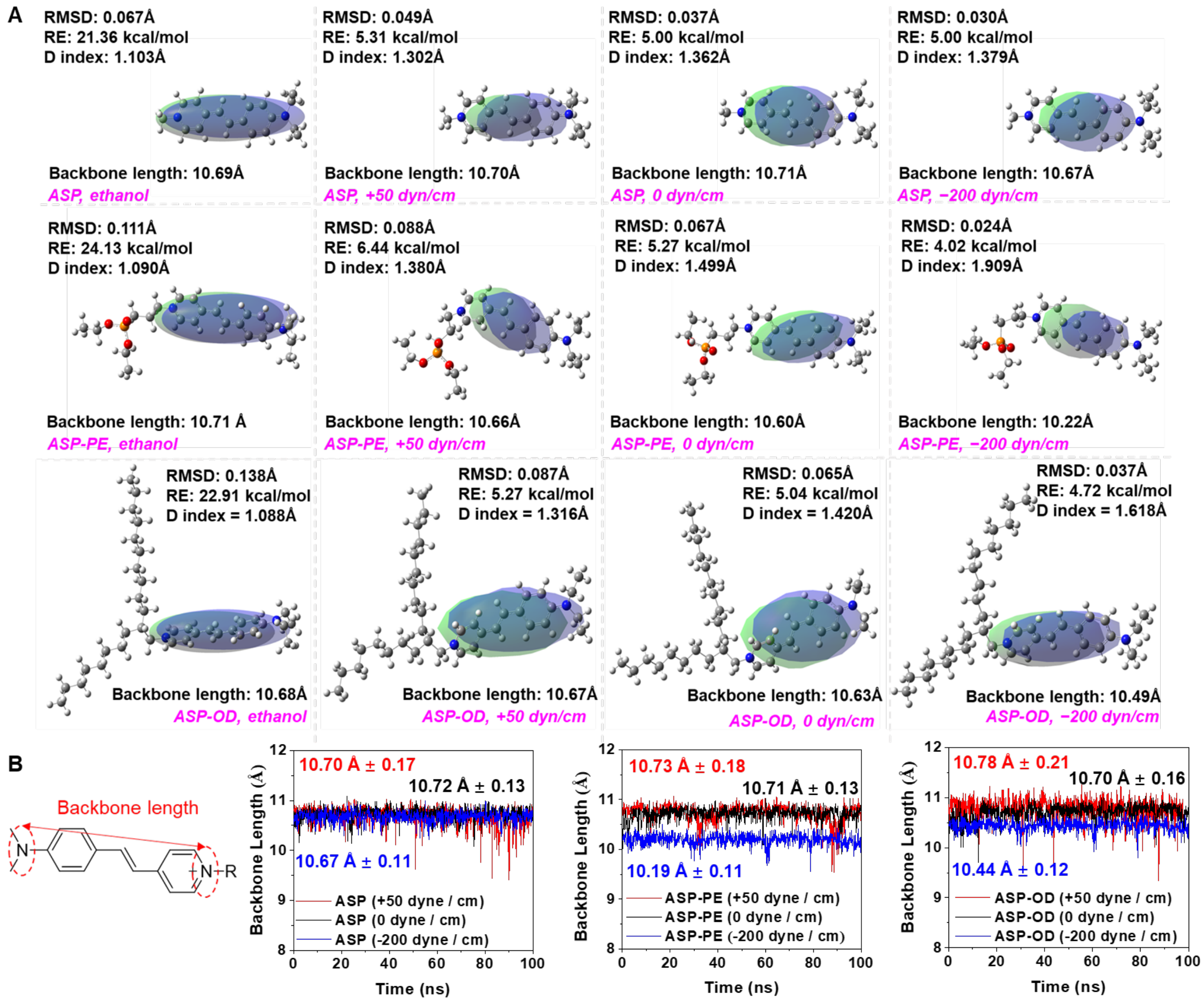

Fig. S20. Simulated molecular properties.

(**A**) Changes in structures (RMSD) and electron densities (D-index) upon excitation and the backbone lengths of ASP, ASP-PE and ASP-OD molecules (top to bottom) in different environments (from left to right, increasing degree of confinement towards intramolecular motions), as well as their total reorganization energies (RE) upon electronic emission. The RMSD were used to evaluate the structural changes between the ground state (S_0 state) and the first single excited state (S_1 state), the D-index were used to evaluate the degree of electron transfer upon electronic excitation, and the RE were used to evaluate the degree of the non-radiative transitions. (**B**) The definition of the backbone length of the styryl dyes; backbone length changes of ASP, ASP-PE and ASP-OD molecules along the simulation time at different positions from the center of the phospholipid bilayers with different membrane tensions (+50, 0 and −200 dyn/cm). The backbone length was calculated used by the MD trajectories of umbrella sampling at the windows where the ASP, ASP-PE and ASP-OD molecules had the most distributions (fig. S19).

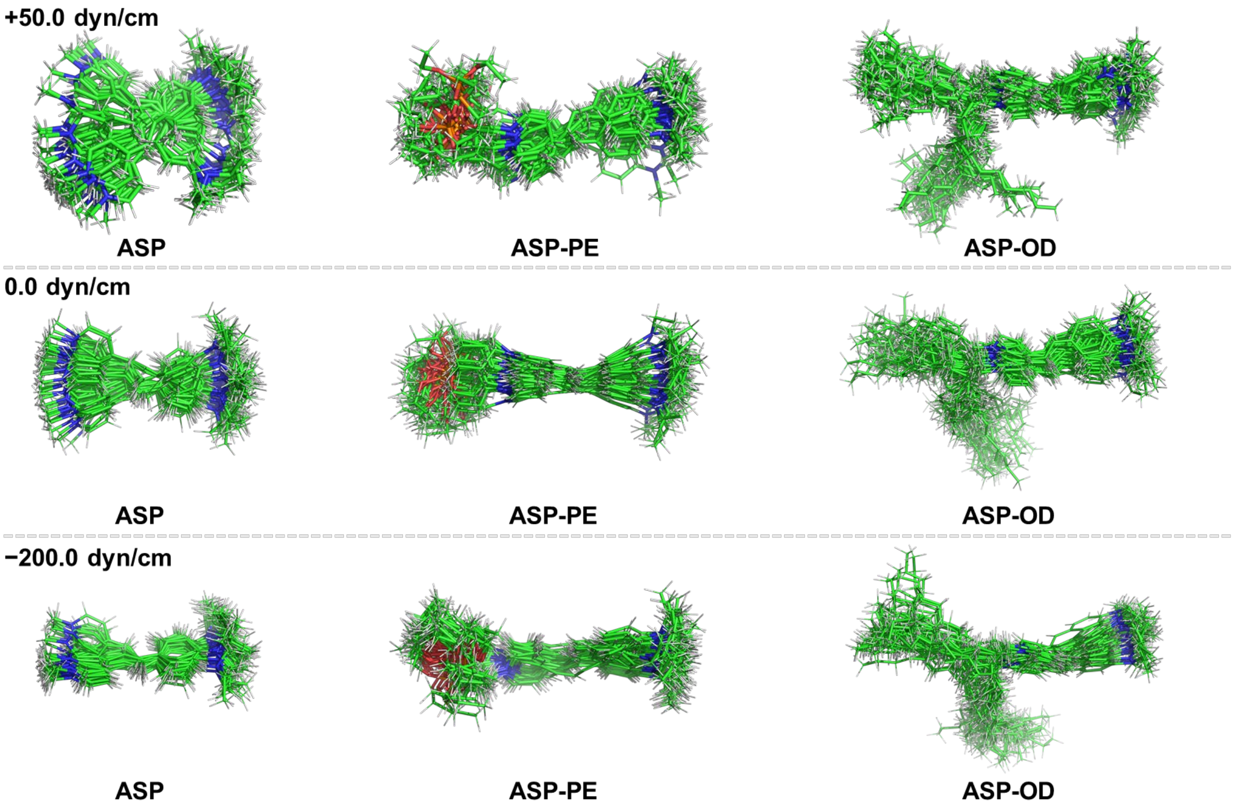

Fig. S21. Superimposed 50 conformations of ASP, ASP-PE, and ASP-OD molecules in the phospholipid membrane with different surface tensions.

The conformations were extracted from the MD trajectories of umbrella sampling at the windows where the ASP, ASP-PE and ASP-OD molecules had the most distributions (see fig. S19).

REFERENCES AND NOTES

1. P. K. Chaudhuri, B. C. Low, C. T. Lim, Mechanobiology of tumor growth. *Chem. Rev.* **118**, 6499–6515 (2018).

2. M. Aragona, T. Panciera, A. Manfrin, S. Giulitti, F. Michielin, N. Elvassore, S. Dupont, S. Piccolo, A mechanical checkpoint controls multicellular growth through YAP/TAZ regulation by actin-processing factors. *Cell* **154**, 1047–1059 (2013).

3. G. R. DiResta, S. S. Nathan, M. W. Manoso, J. Casas-Ganem, C. Wyatt, T. Kubo, P. J. Boland, E. A. Athanasian, J. Miodownik, R. Gorlick, J. H. Healey, Cell proliferation of cultured human cancer cells are affected by the elevated tumor pressures that exist in vivo. *Ann. Biomed. Eng.* **33**, 1270–1280 (2005).

4. J. Das, S. Maji, T. Agarwal, S. Chakraborty, T. K. Maiti, Hemodynamic shear stress induces protective autophagy in HeLa cells through lipid raft-mediated mechanotransduction. *Clin. Exp. Metastasis* **35**, 135–148 (2018).

5. J. Lee, A. A. Abdeen, K. L. Wycislo, T. M. Fan, K. A. Kilian, Interfacial geometry dictates cancer cell tumorigenicity. *Nat. Mater.* **15**, 856–862 (2016).

6. J. Lee, A. A. Abdeen, Y. Li, S. Goonetilleke, K. A. Kilian, Gradient and dynamic hydrogel materials to probe dynamics in cancer stem cell phenotypes. *ACS Appl. Bio Mater.* **4**, 711–720 (2021).

7. J. J. Northey, L. Przybyla, V. M. Weaver, Tissue force programs cell fate and tumor aggression. *Cancer Discov.* **7**, 1224–1237 (2017).

8. A. Diz-Muñoz, D. A. Fletcher, O. D. Weiner, Use the force: Membrane tension as an organizer of cell shape and motility. *Trends Cell Biol.* **23**, 47–53 (2013).

9. A.-L. Le Roux, X. Quiroga, N. Walani, M. Arroyo, P. Roca-Cusachs, The plasma membrane as a mechanochemical transducer. *Philos. Trans. R. Soc. B* **374**, 20180221 (2019).

10. O. Chaudhuri, S. T. Koshy, C. Branco da Cunha, J.-W. Shin, C. S. Verbeke, K. H. Allison, D. J. Mooney, Extracellular matrix stiffness and composition jointly regulate the induction of malignant phenotypes in mammary epithelium. *Nat. Mater.* **13**, 970–978 (2014).

11. Y. Wang, J. Y.-J. Shyy, S. Chien, Fluorescence proteins, live-cell imaging, and mechanobiology: Seeing is believing. *Annu. Rev. Biomed. Eng.* **10**, 1–38 (2008).

12. Y.-L. Zhang, J. A. Frangos, M. Chachisvilis, Laurdan fluorescence senses mechanical strain in the lipid bilayer membrane. *Biochem. Biophys. Res. Commun.* **347**, 838–841 (2006).

13. M. A. Boyd, N. P. Kamat, Visualizing tension and growth in model membranes using optical dyes. *Biophys. J.* **115**, 1307–1315 (2018).

14. L. P. Bharath, J. M. Cho, S.-K. Park, T. Ruan, Y. Li, R. Mueller, T. Bean, V. Reese, R. S. Richardson, J. Cai, A. Sargsyan, K. Pires, P. V. Anandh Babu, S. Boudina, T. E. Graham, J. D. Symons, Endothelial cell autophagy maintains shear stress-induced nitric oxide generation via glycolysis-dependent purinergic signaling to eNOS. *Arterioscler. Thromb. Vasc. Biol.* **37**, 1646–1656 (2017).

15. E. E. Mowers, M. N. Sharifi, K. F. Macleod, Functions of autophagy in the tumor microenvironment and cancer metastasis. *FEBS J.* **285**, 1751–1766 (2018).

16. R. Bretón-Romero, R. Acín-Perez, F. Rodríguez-Pascual, M. Martínez-Molledo, R. P. Brandes, E. Rial, J. A. Enríquez, S. Lamas, Laminar shear stress regulates mitochondrial dynamics, bioenergetics responses and PRX3 activation in endothelial cells. *Biochim Biophys. Acta* **1843**, 2403–2413 (2014).

17. C. Ploumi, I. Daskalaki, N. Tavernarakis, Mitochondrial biogenesis and clearance: A balancing act. *FEBS J.* **284**, 183–195 (2017).

18. A. Colom, E. Derivery, S. Soleimanpour, C. Tomba, M. D. Molin, N. Sakai, M. González-Gaitán, S. Matile, A. Roux, A fluorescent membrane tension probe. *Nat. Chem.* **10**, 1118–1125 (2018).

19. S. Soleimanpour, A. Colom, E. Derivery, M. Gonzalez-Gaitan, A. Roux, N. Sakai, S. Matile, Headgroup engineering in mechanosensitive membrane probes. *Chem. Commun.* **52**, 14450–14453 (2016).

20. A. Goujon, A. Colom, K. Straková, V. Mercier, D. Mahecic, S. Manley, N. Sakai, A. Roux, S. Matile, Mechanosensitive fluorescent probes to image membrane tension in mitochondria, endoplasmic reticulum, and lysosomes. *J. Am. Chem. Soc.* **141**, 3380–3384 (2019).

21. K. Straková, J. López-Andarias, N. Jiménez-Rojo, J. E. Chambers, S. J. Marciniak, H. Riezman, N. Sakai, S. Matile, HaloFlippers: A general tool for the fluorescence imaging of precisely localized membrane tension changes in living cells. *ACS Cent. Sci.* **6**, 1376–1385 (2020).

22. T. C. OwYong, S. Ding, N. Wu, T. Fellowes, S. Chen, J. M. White, W. W. H. Wong, Y. Hong, Optimising molecular rotors to AIE fluorophores for mitochondria uptake and retention. *Chem. Commun.* **56**, 14853–14856 (2020).

23. M.-Y. Wu, A. Y. H. Wong, J.-K. Leung, C. Kam, K. L.-K. Wu, Y.-S. Chan, K. Liu, N. Y. Ip, S. Chen, A near-infrared AIE fluorescent probe for myelin imaging: From sciatic nerve to the optically cleared brain tissue in 3D. *Proc. Natl. Acad. Sci. U.S.A.* **118**, e2106143118 (2021).

24. S. Chen, Y. Hong, Y. Zeng, Q. Sun, Y. Liu, E. Zhao, G. Bai, J. Qu, J. Hao, B. Z. Tang, Mapping live cell viscosity with an aggregation-induced emission fluorogen by means of two-photon fluorescence lifetime imaging. *Chem. A Eur. J.* **21**, 4315–4320 (2015).

25. J. Mei, N. L. C. Leung, R. T. K. Kwok, J. W. Y. Lam, B. Z. Tang, Aggregation-induced emission: Together we shine, united we soar! *Chem. Rev.* **115**, 11718–11940 (2015).

26. L. Magrassi, D. Purves, J. W. Lichtman, Fluorescent probes that stain living nerve terminals. *J. Neurosci.* **7**, 1207–1214 (1987).

27. M. J. McCarthy, J. Baumber, P. H. Kass, S. A. Meyers, Osmotic stress induces oxidative cell damage to rhesus macaque spermatozoa1. *Biol. Reprod.* **82**, 644–651 (2010).

28. K. Yamamoto, J. Ando, Vascular endothelial cell membranes differentiate between stretch and shear stress through transitions in their lipid phases. *Am. J. Physiol. Heart Circ. Physiol.* **309**, H1178–H1185 (2015).

29. M. Páez-Pérez, I. López-Duarte, A. Vyšniauskas, N. J. Brooks, M. K. Kuimova, Imaging non-classical mechanical responses of lipid membranes using molecular rotors. *Chem. Sci.* **12**, 2604–2613 (2021).

30. A. Kostic, C. D. Lynch, M. P. Sheetz, Differential matrix rigidity response in breast cancer cell lines correlates with the tissue tropism. *PLOS ONE* **4**, e6361 (2009).

31. S. Li, F. Zhao, Y. Zhan, X. Liu, T. Hun, H. Zhang, C. Qiu, J. He, Z. Yi, Y. Sun, Y. Fan, How deep might myoblasts sense: The effect of substrate stiffness and thickness on the behavior of myoblasts. *J. Med. Biol. Eng.* **38**, 596–606 (2018).

32. D. E. Discher, P. Janmey, Y.-L. Wang, Tissue cells feel and respond to the stiffness of their substrate. *Science* **310**, 1139–1143 (2005).

33. M. P. Sheetz, J. E. Sable, H.-G. Döbereiner, Continuous membrane-cytoskeleton adhesion requires continuous accommodation to lipid and cytoskeleton dynamics. *Annu. Rev. Biophys. Biomol. Struct.* **35**, 417–434 (2006).

34. J. L. Madara, D. Barenberg, S. Carlson, Effects of cytochalasin D on occluding junctions of intestinal absorptive cells: Further evidence that the cytoskeleton may influence paracellular permeability and junctional charge selectivity. *J. Cell Biol.* **102**, 2125–2136 (1986).

35. M. Schliwa, Action of cytochalasin D on cytoskeletal networks. *J. Cell Biol.* **92**, 79–91 (1982).

36. A. Grossfield, "*WHAM: The weighted histogram analysis method*," version 2.0.11; http://membrane.urmc.rochester.edu/wordpress/?page_id=126.

37. D. Kim, S.-H. Kim, J. Y. Park, Floating-on-water fabrication method for thin polydimethylsiloxane membranes. *Polymers* **11**, 1264 (2019).

38. S. Brasselet, F. Cherioux, P. Audebert, J. Zyss, New octupolar star-shaped strucures for quadratic nonlinear optics. *Chem. Mater.* **11**, 1915–1920 (1999).

39. E. L. Wu, X. Cheng, S. Jo, H. Rui, K. C. Song, E. M. Dávila-Contreras, Y. Qi, J. Lee, V. Monje-Galvan, R. M. Venable, J. B. Klauda, W. Im, CHARMM-GUI membrane builder toward realistic biological membrane simulations. *J. Comput. Chem.* **35**, 1997–2004 (2014).

40. S. Kim, J. Lee, S. Jo, C. L. Brooks III, H. S. Lee, W. Im, CHARMM-GUI ligand reader and modeler for CHARMM force field generation of small molecules. *J. Comput. Chem.* **38**, 1879–1886 (2017).

41. D. A. Case, K. Belfon, I. Y. Ben-Shalom, S. R. Brozell, D. S. Cerutti, T. E. Cheatham III, V. W. D. Cruzeiro, T. A. Darden, R. E. Duke, G. Giambasu, M. K. Gilson, H. Gohlke, A. W. Goetz, R Harris, S. Izadi, S. A. Izmailov, K. Kasavajhala, A. Kovalenko, R. Krasny, T. Kurtzman, T.S. Lee, S. LeGrand, P. Li, C. Lin, J. Liu, T. Luchko, R. Luo, V. Man, K. M. Merz, Y. Miao, O. Mikhailovskii, G. Monard, H. Nguyen, A. Onufriev, F. Pan, S. Pantano, R. Qi, D. R. Roe, A. Roitberg, C. Sagui, S. Schott-Verdugo, J. Shen, C. L. Simmerling, N. R. Skrynnikov, J. Smith, J. Swails, R. C. Walker, J. Wang, L. Wilson, R. M. Wolf, X. Wu, Y. Xiong, Y. Xue, D. M. York, P. A. Kollman, *AMBER 20* (University of California, San Francisco, 2020).

42. W. L. Jorgensen, J. Chandrasekhar, J. D. Madura, R. W. Impey, M. L. Klein, Comparison of simple potential functions for simulating liquid water. *J. Chem. Phys.* **79**, 926–935 (1983).

43. C. J. Dickson, R. C. Walker, I. R. Gould, Lipid21: Complex lipid membrane simulations with AMBER. *J. Chem. Theory Comput.* **18**, 1726–1736 (2022).

44. W. D. Cornell, P. Cieplak, C. I. Bayly, I. R. Gould, K. M. Merz, D. M. Ferguson, D. C. Spellmeyer, T. Fox, J. W. Caldwell, P. A. Kollman, A second generation force field for the simulation of proteins, nucleic acids, and organic molecules. *J. Am. Chem. Soc.* **117**, 5179–5197 (1995).

45. V. Hornak, R. Abel, A. Okur, B. Strockbine, A. Roitberg, C. Simmerling, Comparison of multiple Amber force fields and development of improved protein backbone parameters. *Proteins* **65**, 712–725 (2006).

46. D. Svozil, J. E. Sponer, I. Marchan, A. Pérez, T. E. Cheatham III, F. Forti, F. Javier Luque, M. Orozco, J. Sponer, Geometrical and electronic structure variability of the sugar–phosphate backbone in nucleic acids. *J. Phys. Chem. B* **112**, 8188–8197 (2008).

47. J. Wang, P. Cieplak, P. A. Kollman, How well does a restrained electrostatic potential (RESP) model perform in calculating conformational energies of organic and biological molecules? *J. Comput. Chem.* **21**, 1049–1074 (2000).

48. Gaussian 16, Revision B.01, M. J. Frisch, G. W. Trucks, H.B. Schlegel, G. E. Scuseria, M. A. Robb, J. R. Cheeseman, G. Scalmani, V. Barone, G. A. Petersson, H. Nakatsuji, X. Li, M. Caricato, A. V. Marenich, J. Bloino, B. G. Janesko, R. Gomperts, B. Mennucci, H. P. Hratchian, Ortiz, J. V., A. F. Izmaylov, J. L. Sonnenberg, D. Williams-Young, F. Ding, F. Lipparini, F. Egidi, J. Goings, B. Peng, A. Petrone, T. Henderson, D. Ranasinghe, V. G. Zakrzewski, J. Gao, N. Rega, G. Zheng, W. Liang, M. Hada, M. Ehara, K. Toyota, R. Fukuda, J. Hasegawa, M. Ishida, T. Nakajima, Y. Honda, O. Kitao, H. Nakai, T. Vreven, K. Throssell, J. A. Montgomery Jr., J. E. Peralta, F. Ogliaro, M. J. Bearpark, J. J. Heyd, E. N. Brothers, K. N. Kudin, V. N. Staroverov, T. A. Keith, R. Kobayashi, J. Normand, K. Raghavachari, A. P. Rendell, J. C. Burant, S. S. Iyengar, J. Tomasi, M. Cossi, J. M. Millam, M. Klene, C. Adamo, R. Cammi, J. W. Ochterski, R. L. Martin, K. Morokuma, O. Farkas, J. B. Foresman, D. J. Fox, *GaussView 5.0.* (Gaussian Inc., 2016).

49. X. Wu, B. R. Brooks, Self-guided Langevin dynamics simulation method. *Chem. Phys. Lett.* **381**, 512–518 (2003).

50. J.-P. Ryckaert, G. Ciccotti, H. J. C. Berendsen, Numerical integration of the cartesian equations of motion of a system with constraints: Molecular dynamics of *n*-alkanes. *J. Comput. Phys.* **23**, 327–341 (1977).

51. G. M. Torrie, J. P. Valleau, Nonphysical sampling distributions in Monte Carlo free-energy estimation: Umbrella sampling. *J. Comput. Phys.* **23**, 187–199 (1977).

52. R. Vijayaraj, S. Van Damme, P. Bultinck, V. Subramanian, Molecular dynamics and umbrella sampling study of stabilizing factors in cyclic peptide-based nanotubes. *J. Phys. Chem. B* **116**, 9922–9933 (2012).

53. S. Kumar, J. M. Rosenberg, D. Bouzida, R. H. Swendsen, P. A. Kollman, THE weighted histogram analysis method for free-energy calculations on biomolecules. I. The method. *J. Comput. Chem.* **13**, 1011–1021 (1992).

54. Extension to the weighted histogram analysis method: Combining umbrella sampling with free energy calculations. *Comput. Phys. Commun.* **135**, 40–57 (2001).

55. C. T. Lee, J. Comer, C. Herndon, N. Leung, A. Pavlova, R. V. Swift, C. Tung, C. N. Rowley, R. E. Amaro, C. Chipot, Y. Wang, J. C. Gumbart, Simulation-based approaches for determining membrane permeability of small compounds. *J. Chem. Inf. Model.* **56**, 721–733 (2016).

56. G. Hummer, Position-dependent diffusion coefficients and free energies from Bayesian analysis of equilibrium and replica molecular dynamics simulations. *New J. Phys.* **7**, 34–34 (2005).

57. G. Scalmani, M. J. Frisch, Continuous surface charge polarizable continuum models of solvation. I. General formalism. *J. Chem. Phys.* **132**, 114110 (2010).

58. S. Dapprich, I. Komáromi, K. S. Byun, K. Morokuma, M. J. Frisch, A new ONIOM implementation in Gaussian98. Part I. The calculation of energies, gradients, vibrational frequencies and electric field derivatives. *J. Mol. Struct. Theochem* **461**, 1–21 (1999).

59. O. A. Vydrov, G. E. Scuseria, Assessment of a long-range corrected hybrid functional. *J. Chem. Phys.* **125**, 234109 (2006).

60. L. A. Curtiss, M. P. McGrath, J.P. Blaudeau, N. E. Davis, R. C. Binning Jr, L. Radom, Extension of Gaussian-2 theory to molecules containing third-row atoms Ga–Kr. *J. Chem. Phys.* **103**, 6104–6113 (1995).

61. J. Andzelm, E. Wimmer, Density functional Gaussian-type-orbital approach to molecular geometries, vibrations, and reaction energies. *J. Chem. Phys.* **96**, 1280–1303 (1992).

62. M. Petersilka, U. J. Gossmann, E. K. U. Gross, Excitation energies from time-dependent density-functional theory. *Phys. Rev. Lett.* **76**, 1212–1215 (1996).

63. R. C. Hilborn, Einstein coefficients, cross sections,f values, dipole moments, and all that. *Am. J. Phys.* **50**, 982–986 (1982).

64. Y. Niu, W. Li, Q. Peng, H. Geng, Y. Yi, L. Wang, G. Nan, D. Wang, Z. Shuai, MOlecular MAterials Property Prediction Package (MOMAP) 1.0: A software package for predicting the luminescent properties and mobility of organic functional materials. *Mol. Phys.* **116**, 1078–1090 (2018).

65. Q. Peng, Y. Yi, Z. Shuai, J. Shao, Toward quantitative prediction of molecular fluorescence quantum efficiency: Role of Duschinsky rotation. *J. Am. Chem. Soc.* **129**, 9333–9339 (2007).

66. Z. Shuai, Q. Peng, Excited states structure and processes: Understanding organic light-emitting diodes at the molecular level. *Phys. Rep.* **537**, 123–156 (2014).

67. Z. Shuai, Q. Peng, Organic light-emitting diodes: Theoretical understanding of highly efficient materials and development of computational methodology. *Natl. Sci. Rev.* **4**, 224–239 (2017).

68. S. Braslavsky, Glossary of terms used in photochemistry 3rd edition (IUPAC Recommendations 2006). *Pure Appl. Chem.* **79**, 293–465 (2007).
